# Association Between Endometriosis and Cardiovascular Disease: Insights From the UK Biobank

**DOI:** 10.1161/JAHA.125.048454

**Published:** 2026-07-10

**Authors:** Clara M. Meijs, Benjamin Gruenberger, Sanne A. E. Peters, Yvonne T. van der Schouw, N. Charlotte Onland‐Moret, Sunge Makawa, Lisa Seekircher, Anna Lena Zippl, Beata Seeber, Josef Fritz, Peter Willeit, Lena Tschiderer

**Affiliations:** ^1^ Institute of Clinical Epidemiology, Public Health, Health Economics, Medical Statistics and Informatics, Medical University of Innsbruck Innsbruck Austria; ^2^ Julius Center for Health Sciences and Primary Care University Medical Center Utrecht, Utrecht University Utrecht The Netherlands; ^3^ The George Institute for Global Health, School of Public Health, Imperial College London London UK; ^4^ Department of Gynecologic Endocrinology and Reproductive Medicine Medical University Innsbruck Innsbruck Austria; ^5^ Department of Translational Medicine Lund University Malmö Sweden; ^6^ Department of Public Health and Primary Care University of Cambridge Cambridge UK; ^7^ Ignaz Semmelweis Institute, Interuniversity Institute for Infection Research, Medical University of Vienna Vienna Austria

**Keywords:** cardiovascular disease, endometriosis, hysterectomy, oophorectomy, UK Biobank, Cardiovascular Disease, Women, Epidemiology

## Abstract

**Background:**

Endometriosis has been associated with a higher risk of cardiovascular disease (CVD), but the underlying mechanisms remain incompletely understood.

**Methods:**

Using UK Biobank data, we assessed the relationship between endometriosis and CVD (defined as a composite of fatal and non‐fatal coronary heart disease, peripheral artery disease, and stroke), cardiovascular mortality, and all‐cause mortality, applying Cox regression analysis while adjusting for conventional and emerging, lifestyle and demographic, and female‐specific CVD risk factors. Furthermore, we performed subgroup analysis across age and female‐specific factors, and mediation analysis to study the role of hysterectomy and oophorectomy.

**Results:**

We included 264 740 women without CVD at baseline (median age 57 [25th–75th percentile 50–63] years; 8194 with endometriosis) from the UK Biobank. During 3 484 788 person‐years of follow‐up, 22 283 women developed CVD. Endometriosis was associated with an 18% higher risk of CVD (hazard ratio 1.18 [95% CI 1.10–1.28]), mainly driven by coronary heart disease (1.24 [1.12–1.37]). Conversely, endometriosis was related to a 12% lower risk of all‐cause mortality (0.88 [0.80–0.98]). The association with cardiovascular mortality was not significant (0.85 [0.63–1.13]). Mediation analysis suggested that hysterectomy may partially mediate the association between endometriosis and CVD. Subgroup analyses did not yield significant differences.

**Conclusions:**

Endometriosis is associated with a higher risk of CVD, especially coronary heart disease. Mediation analysis suggests that hysterectomy partly explains this association. Whether hysterectomy may proxy endometriosis severity instead of being a true mediator remains to be elucidated. These findings highlight the need for studies that unravel the causal mechanisms linking endometriosis, its treatments, and cardiovascular outcomes.

## Graphical abstract

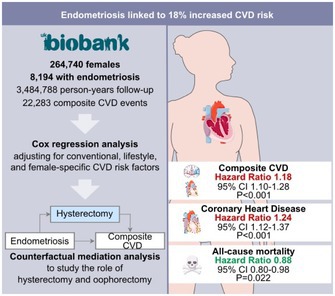

**Nonstandard Abbreviations and Acronyms**
OCP: oral contraceptive pill
UKB: UK Biobank

**Research Perspective**

**What is New?**

- This study included >250 000 female individuals from the UK Biobank and showed that endometriosis is associated with an 18% higher risk of cardiovascular disease, especially coronary heart disease.
- Our mediation analysis indicates that hysterectomy, with or without oophorectomy, accounts for part of the observed endometriosis cardiovascular disease association; nevertheless, it is unclear whether hysterectomy represents a true mediating pathway or simply serves as a marker of endometriosis severity.

**What Question Should Be Addressed Next?**

- Further research is needed to clarify the causal pathways between endometriosis, its treatments, and cardiovascular outcomes.

Cardiovascular disease (CVD) is the leading cause of global disease burden and mortality.^1^ Historically, CVD in women has been under‐recognized and under‐studied, although the incidence of CVD is similar in women and men.^2^ To develop a tailored approach for the prevention and treatment of the disease, it is crucial to understand sex‐specific risk factors for CVD. Several female‐specific factors such as age at menarche, adverse pregnancy outcomes (eg preeclampsia, gestational diabetes), early menopause, and polycystic ovary syndrome have been linked to CVD risk.^3^

Another female‐specific factor gaining attention is endometriosis, a chronic gynecological disorder defined by the presence of endometrial‐like tissue outside the uterus, leading to pain and infertility, affecting approximately 5%–10% of women of reproductive age.^4^ Once considered a condition confined to the pelvis, endometriosis is now recognized as a systemic disease that disrupts metabolic processes in the liver and adipose tissue and promotes inflammation.^4^ A range of meta‐analyses of observational studies linked endometriosis to a higher risk of ischemic heart disease, cerebrovascular events, peripheral artery disease, and major adverse cardiovascular events.^5^, ^6^, ^7^, ^8^ Women with endometriosis also have elevated risks of hypertension and hypercholesterolemia, both known contributors to CVD.^9^, ^10^

Despite growing evidence, the link between endometriosis and CVD remains incompletely understood. Leading hypotheses point to chronic systemic inflammation, accelerated atherogenesis, and metabolic dysregulation as key mechanisms underlying the relationship between endometriosis and CVD. Inflammation, a hallmark of both endometriosis and atherosclerosis, promotes vascular damage, atherosclerotic plaque formation, and microvascular dysfunction.^5^, ^9^, ^11^, ^12^, ^13^ Although most studies on endometriosis and CVD have not adequately considered surgical treatments, the few that have done so suggest that both oophorectomy and hysterectomy play a mediating role in the association.^14^, ^15^, ^16^

This study aims to clarify the link between endometriosis and CVD and distinguish potential effects of hysterectomy with or without oophorectomy from biologically driven associations, using mediation analysis within the large and detailed longitudinal data from the UK Biobank (UKB).

## METHODS

The UK Biobank data used in this study are available upon request via the UKB website (https://www.ukbiobank.ac.uk/enable‐your‐research/apply‐for‐access). We do not have permission to share or redistribute the data used in this study, as access is governed by the UK Biobank. Researchers can obtain the same data by submitting a research application and gaining approval from UK Biobank.

The results of this study are presented in accordance with the STROBE guidelines. The STROBE checklist is available in Table S1.

### Data Source

This study used data from the UKB, a large‐scale prospective cohort that enrolled over 500 000 individuals aged 40 to 69 years across the United Kingdom between 2006 and 2010.^17^, ^18^ The date of recruitment is referred to as baseline throughout the manuscript. Participants underwent comprehensive baseline assessments at one of 22 centers, which included questionnaires, physical examinations, and biological sample collection. The UKB has ethical approval from the North West Multi‐Centre Research Ethics Committee, and all participants gave written informed consent.

### Study Population

Individuals who identified as female at baseline were included. Participants with a history of CVD (defined by *International Classification of Diseases, Tenth Revision [ICD‐10]* codes I20–I25, I60–I69, and I73.8–I73.9) before recruitment were excluded.

### Definition of Endometriosis and CVD End Points

Both endometriosis and cardiovascular outcomes were defined using clinical coding systems from linked health records, including hospital admission data, death records (*ICD, Ninth Revision* [*ICD‐9*] and *ICD‐10*), and general practitioner records (Read codes v2 and CTV3). Definitions were based on the UK Biobank “first occurrence of disease” framework, which maps across coding systems to standardize case ascertainment using *ICD‐10* codes.^19^

Endometriosis was defined as the documentation of an *ICD‐10* N80 diagnosis in linked health records from either general practitioners or hospital admissions at baseline, or if the participant self‐reported having endometriosis at baseline.

The primary CVD end point was defined as composite CVD, including *ICD‐10* codes I21–I25 (coronary heart disease [CHD]), I60–I69 (stroke), I73.8–I73.9 (peripheral artery disease [PAD]), and revascularization (K41–K46, K49, K50.1, 50.2, K50.4, K75). Secondary end points included CHD (I21–I25), myocardial infarction (MI) (I21, I22), PAD (I73.8–I73.9), stroke (I60–I69), ischemic stroke (I63, I64), hemorrhagic stroke (I60, I61), cardiovascular mortality (death with an underlying cause coded as I05–I28, I30–I52, I60–I82), and all‐cause mortality (any fatal event). The end of follow‐up was set at December 31, 2022. Time‐to‐event for cardiovascular events was defined as the time from baseline to the first cardiovascular event, end of follow‐up, loss to follow‐up, or death, whichever occurred first. For mortality outcomes, time‐to‐event was defined as the time from baseline to end of follow‐up, loss to follow‐up, or death, whichever occurred first. If multiple cardiovascular events occurred on the same date, a hierarchical classification was applied to assign a single outcome: CHD was prioritized over stroke, stroke was prioritized over PAD, and PAD was prioritized over revascularization. Among strokes, ischemic strokes were prioritized over hemorrhagic strokes. The definition of the covariates is described in Data S1.

### Statistical Analysis

All analyses were performed using R Statistical Software (v4.4.1).^20^ All tests were 2‐sided, and *P* values of 0.05 or less were considered statistically significant. Details on the handling of missing values are provided in Data S1.

#### Descriptive Statistics

Descriptive statistics are reported as mean and standard deviation for normally distributed continuous variables, median and 25th to 75th percentile for non‐normally distributed continuous variables and counts with percentages for categorical variables. Distribution of continuous variables was assessed using histograms.

#### Primary Analysis

The association between endometriosis and CVD was estimated by performing Cox regression with attained age as the underlying time scale to estimate hazard ratios (HRs) and 95% CIs. There was no evidence of violation of the proportional hazards assumption for endometriosis based on Schoenfeld residual tests (*P*>0.05), supported by visual inspection of Schoenfeld and log–log plots (Figure S1). We used a single model that adjusted for conventional and emerging cardiovascular risk factors (body mass index, smoking status, diabetes, low‐density lipoprotein cholesterol, systolic blood pressure, lipoprotein(a)), lifestyle and demographic variables (alcohol use, physical activity, ethnicity, Townsend Deprivation Index), and female‐specific risk factors (age at menarche, ever oral contraceptive pill [OCP] use). Other female‐specific CVD risk factors like parity, history of polycystic ovary syndrome, menopausal status, ever HRT use, and history of stillbirth are included in the subgroup analysis since we suspect they might be effect modifiers in the endometriosis‐CVD relationship but no confounders. The primary end point was composite CVD, and models for all secondary end points (CHD, stroke, PAD, cardiovascular mortality, and all‐cause mortality) were also constructed.

#### Subgroup Analysis

We conducted subgroup analyses by including interaction terms between endometriosis and each subgroup variable of interest (history of hysterectomy, history of oophorectomy, history of hysterectomy or oophorectomy, ever OCP use, ever HRT use, menopausal status, history of polycystic ovary syndrome, age at baseline [continuous for interaction term, and categorized in <60 or ≥60 years for subgroup HRs], parity, history of stillbirth, and history of miscarriage) in the Cox regression models. Each model included endometriosis, the subgroup variable of interest, and their interaction term, while adjusting for all variables specified in the primary analysis. The primary end point (composite CVD) was used for all analyses.

#### Mediation Analysis

We examined whether the association between endometriosis and composite CVD was mediated by gynecologic surgery using a counterfactual‐based weighting approach.^21^ Three mediators were evaluated: history of (1) hysterectomy without oophorectomy, (2) hysterectomy with oophorectomy, and (3) hysterectomy with or without oophorectomy. Mediation analysis was performed using the *mets* R‐package (v1.3.5).^22^ Stabilized weights were derived from fully adjusted logistic regression models predicting each mediator and used in fully adjusted weighted Cox regression models with age as time scale to estimate direct, indirect, and total effects. HRs and 95% CIs were computed, and the proportion mediated was calculated as the log ratio of indirect to total effect. CIs and *P* values were derived using the delta method under the assumption of independence. To investigate temporal ordering, median and 25th to 75th percentile of latencies between endometriosis diagnosis and hysterectomy, and between hysterectomy and the composite CVD event, were examined.

#### Sensitivity Analysis

Several sensitivity analyses were performed to assess the robustness of the findings of our primary analysis, each using fully adjusted Cox proportional hazards models and composite CVD as the end point, unless stated otherwise. First, to assess the impact of the adjustment, we constructed three additional models with increasing levels of confounder control. Model 1 was unadjusted; Model 2 adjusted for conventional and emerging cardiovascular risk factors; and Model 3 further included lifestyle and demographic variables. Second, we restricted analyses to non‐self‐reported endometriosis to evaluate potential exposure misclassification. Third, to reduce reverse causation and ensure correct temporal ordering, we performed a sensitivity analysis where we repeated the primary analysis and mediation analysis, this time only including endometriosis diagnoses first recorded before hysterectomy. Endometriosis cases diagnosed during or after hysterectomy may reflect incidental findings, potentially biasing mediation estimates by overrepresenting surgically detected cases. Fourth, we additionally performed fully adjusted classical mediation analysis,^23^ also known as difference‐in‐coefficients method, to investigate the stability of the results. Fifth, to examine the influence of adenomyosis, we repeated the analysis excluding women with an adenomyosis diagnosis from the endometriosis group. Sixth, a complete case analysis was performed, restricting the sample to participants without missing data, to evaluate the impact of imputation on the results. Seventh, the mediation analysis was repeated in strata defined by ever HRT use, to evaluate potential effect modification. Eight, we repeated the primary analysis using time‐to‐event as timescale and age as an additional covariate. Ninth, we repeated the primary analysis by censoring follow‐up 3 years after endometriosis diagnosis to account for the window of opportunity. Tenth, we performed competing risks regression using the Fine–Gray subdistribution hazard model to account for non‐CVD mortality as a competing event.^24^

In an additional sensitivity analysis, we aimed to inspect whether the association between hysterectomy and CVD was independent of endometriosis. Therefore, we investigated the association between (1) hysterectomy (with and without oophorectomy) and (2) hysterectomy without oophorectomy and composite CVD by performing a fully adjusted Cox regression analysis while stratifying women on endometriosis status.

## RESULTS

### Study Population

**Figure 1:**
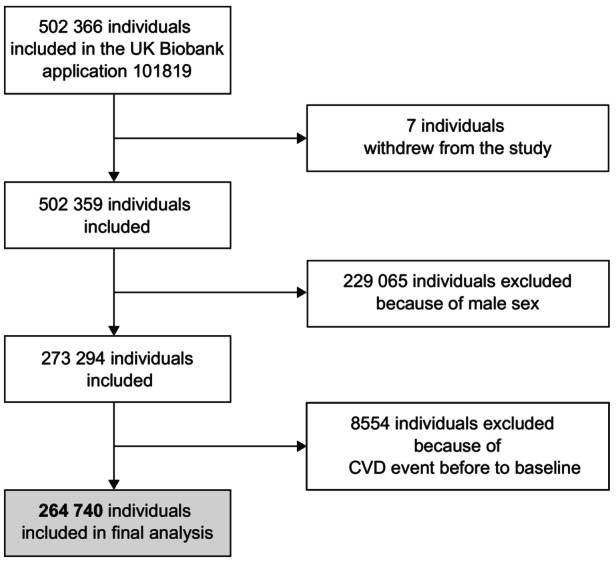
Flowchart of participant inclusion and exclusion. This diagram outlines the selection process of study participants, including reasons for exclusion at each stage. It shows the number of individuals assessed for eligibility, excluded based on predefined criteria, and ultimately included in the final analysis. CVD event before baseline means participants with a history of cardiovascular events (defined as *ICD* codes I20–I25, I60–I69 and I73.8–I73.9) before recruitment. CVD indicates cardiovascular disease.

Baseline characteristics of the study population are presented in the Table 1. Of the 502 366 participants eligible for inclusion in the UKB, 264 740 were women and had no history of CVD at baseline and were thus included in the analysis (Figure 1). Among the participants, 8194 cases of endometriosis were reported. The median age at baseline was 57 years (25th‐75th percentile: 50–63), and 75% of women were postmenopausal. At baseline, women with endometriosis compared with those without endometriosis were younger (median age 53 versus 57 years), more likely to already be in menopause (80% versus 75%), have had a hysterectomy (58% versus 17%), or oophorectomy (37% versus 7%) or have ever used OCPs in the past (86% versus 81%).

**Table 1:** Baseline Characteristics

	Total (N=264 740)	No endometriosis (N=256 546)	Endometriosis (N=8194)	Percentage of missing values
General characteristics				
Age at baseline (y)	57 [50, 63]	57 [50, 63]	53 [47, 60]	0%
Race or ethnicity				0.5%
White	249 340 (94.6%)	241 598 (94.6%)	7742 (94.8%)	
Asian	5372 (2.0%)	5223 (2.0%)	149 (1.8%)	
Black	4480 (1.7%)	4347 (1.7%)	133 (1.6%)	
Other or multiple ethnicities	4328 (1.6%)	4187 (1.6%)	141 (1.7%)	
Townsend deprivation index	−2.2 [−3.6, 0.4]	−2.2 [−3.7, 0.4]	−2.2 [−3.6, 0.4]	0.1%
Follow‐up time (y)	13.7 [12.9, 14.5]	13.7 [12.9, 14.5]	13.7 [13.0, 14.4]	0%
CVD risk factors				
Systolic blood pressure (mm Hg)	135.1 (19.2)	135.2 (19.2)	133.0 (18.6)	6.0%
Diastolic blood pressure (mmHg)	80.7 (10.0)	80.7 (10.0)	80.9 (10.0)	6.0%
Body mass index (kg/m^2^)	27.0 (5.2)	27.0 (5.2)	27.4 (5.2)	0.5%
LDL cholesterol (mmol/L)	3.6 (0.9)	3.6 (0.9)	3.6 (0.9)	7.0%
Lipoprotein(a) (nmol/L)	22.2 [9.9, 61.9]	22.2 [9.9, 61.9]	20.9 [9.6, 61.8]	25.1%
Smoking status				0.5%
Never	158 035 (60.0%)	153 131 (60.0%)	4904 (60.1%)	
Previous	82 114 (31.2%)	79 666 (31.2%)	2448 (30.0%)	
Current	23 161 (8.8%)	22 354 (8.8%)	807 (9.9%)	
Alcohol use frequency				0.3%
Never	24 427 (9.3%)	23 666 (9.2%)	761 (9.3%)	
Special occasions only	39 298 (14.9%)	38 038 (14.9%)	1260 (15.4%)	
One to three times a month	34 414 (13.0%)	33 148 (13.0%)	1266 (15.5%)	
Once or twice a week	68 301 (25.9%)	66 135 (25.8%)	2166 (26.5%)	
Three or four times a week	54 751 (20.7%)	53 139 (20.8%)	1612 (19.7%)	
Daily or almost daily	42 840 (16.2%)	41 727 (16.3%)	1113 (13.6%)	
History of diabetes				0.4%
No	254 420 (96.5%)	246 520 (96.5%)	7900 (96.8%)	
Yes	9179 (3.5%)	8915 (3.5%)	264 (3.2%)	
Physical activity (IPAQ category)				26.2%
Low	35 083 (18.0%)	33 867 (17.9%)	1216 (20.0%)	
Moderate	83 520 (42.8%)	81 017 (42.9%)	2503 (41.1%)	
High	76 521 (39.2%)	74 156 (39.2%)	2365 (38.9%)	
Female‐specific factors				
Age at menarche (y)	13.0 (1.6)	13.0 (1.6)	12.8 (1.7)	3.2%
Ever OCP use				0.5%
No	49 246 (18.7%)	48 088 (18.8%)	1158 (14.2%)	
Yes	214 146 (81.3%)	207 151 (81.2%)	6995 (85.8%)	
Ever HRT use				0.6%
No	163 868 (62.2%)	160 014 (62.7%)	3854 (47.3%)	
Yes	99 382 (37.8%)	95 093 (37.3%)	4289 (52.7%)	
Postmenopausal				4.6%
No	63 024 (25.0%)	61 424 (25.1%)	1600 (20.5%)	
Yes	189 540 (75.0%)	183 338 (74.9%)	6202 (79.5%)	
Parity				0.3%
0	49 925 (18.9%)	47 664 (18.6%)	2261 (27.7%)	
1	35 305 (13.4%)	33 903 (13.3%)	1402 (17.1%)	
2	115 722 (43.8%)	112 594 (44.0%)	3128 (38.3%)	
≥3	62 995 (23.9%)	61 611 (24.1%)	1384 (16.9%)	
History of stillbirth				1.9%
No	258 153 (97.5%)	250 134 (97.5%)	8019 (97.9%)	
Yes	6587 (2.5%)	6412 (2.5%)	175 (2.1%)	
History of spontaneous miscarriage				2.0%
No	205 451 (79.2%)	199 266 (79.3%)	6185 (77.3%)	
Yes	53 966 (20.8%)	52 151 (20.7%)	1815 (22.7%)	
History of adenomyosis				0%
No	263 105 (99.4%)	256 546 (100.0%)	6559 (80.0%)	
Yes	1635 (0.6%)	0 (0.0%)	1635 (20.0%)	
History of PCOS				0%
No	263 759 (99.6%)	255 667 (99.7%)	8092 (98.8%)	
Yes	981 (0.4%)	879 (0.3%)	102 (1.2%)	
History of hysterectomy				0.4%
No	215 400 (81.7%)	211 965 (82.9%)	3435 (42.1%)	
Yes	48 380 (18.3%)	43 649 (17.1%)	4731 (57.9%)	
History of oophorectomy				1.6%
No	239 753 (92.0%)	234 706 (93.0%)	5047 (63.1%)	
Yes	20 722 (8.0%)	17 770 (7.0%)	2952 (36.9%)	

Data are presented as mean (SD), median [25th, 75th percentile], or count (percentage).

BMI indicates body mass index; CVD, cardiovascular disease; HRT, hormone replacement therapy; IPAQ, International Physical Activity Questionnaire; kg/m^2^, kilograms per square meter; LDL, low‐density lipoprotein; mm Hg, millimeters of mercury; mmol/L, millimoles per liter; N, number of women; nmol/L, nanomoles per liter; OCP, oral contraceptive pill; and PCOS, polycystic ovary syndrome.

### Primary Analysis

**Figure 2:**
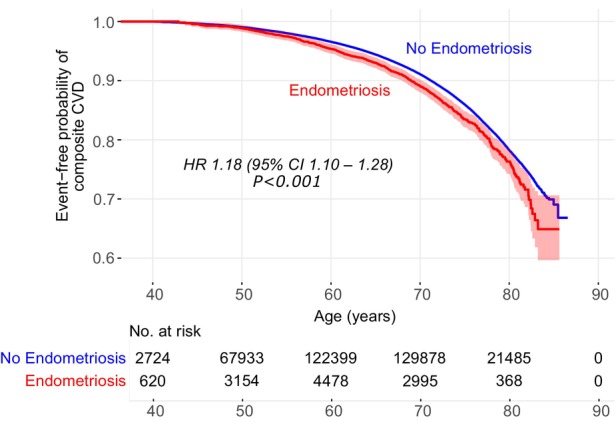
Kaplan–Meier Curve on endometriosis and composite CVD. Kaplan–Meier curves showing the event‐free probability of composite CVD in participants with and without endometriosis. The shaded areas represent 95% CIs. Time was calculated from age at study recruitment to the age of first cardiovascular event, censoring, or end of follow‐up. Numbers at risk are shown below the plots. The curves are unadjusted; HR of the association between endometriosis and composite CVD were evaluated using a fully adjusted Cox proportional hazards model from the primary analysis, adjusting for BMI, smoking status, diabetes, low‐density lipoprotein cholesterol, systolic blood pressure, lipoprotein(a), alcohol use, physical activity, ethnicity, the Townsend Deprivation Index, age at menarche, and ever oral contraceptive pill use. CVD, cardiovascular disease; and HR, hazard ratio.

**Figure 3:**
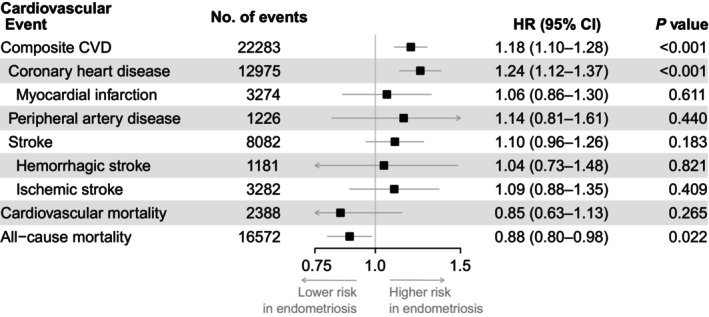
Associations between endometriosis and cardiovascular and all‐cause mortality outcomes. Forest plot shows the association between endometriosis and cardiovascular events. This plot displays hazard ratios with 95% CIs from Cox proportional hazards models with full adjustment and attained age as timescale. Full adjustment includes conventional and emerging CVD risk factors (BMI, smoking status, diabetes, low‐density lipoprotein cholesterol, systolic blood pressure, and lipoprotein(a)), lifestyle and demographic factors (alcohol use, physical activity, ethnicity, and the Townsend Deprivation Index), and female‐specific factors (age at menarche and ever OCP use). CVD, cardiovascular disease; and HR, hazard ratio.

During 3 484 788 person‐years of follow‐up and a median follow‐up time of 13.7 years (25th–75th percentile: 12.9–14.5), 22 283 women experienced a fatal or non‐fatal composite CVD event. Specifically, 12 975 CHDs (with 3274 MIs), 8082 strokes (1181 hemorrhagic strokes, 3282 ischemic strokes), and 1226 PAD cases were observed. Additionally, 16 572 women experienced a fatal event, of which 2388 (14.4%) were fatal cardiovascular events. Results of the fully adjusted primary analysis are depicted in Figure 2. Women with endometriosis showed a higher risk for composite CVD (fully adjusted HR 1.18 [95% CI 1.10–1.28], *P*<0.001). As shown in Figure 3, the higher risk for the composite CVD end point was primarily driven by a statistically significantly higher risk for CHD (fully adjusted HR 1.24 [95% CI 1.12–1.37], *P*<0.001). Risk of all‐cause mortality was significantly lower in women with endometriosis (fully adjusted HR 0.88 [95% CI 0.80–0.98]). The associations between endometriosis and risks for MI, PAD, stroke, ischemic stroke, hemorrhagic stroke, and cardiovascular mortality were not statistically significant (all *P>*0.05).

### Subgroup Analysis

Subgroup analyses showed that the association between endometriosis and CVD did not differ across any of the predefined subgroups including age at baseline, history of hysterectomy, history of oophorectomy, history of hysterectomy or oophorectomy, menopausal status, ever HRT use, polycystic ovary syndrome, ever OCP use, parity, history of stillbirth, or history of spontaneous miscarriage (all *P*>0.05; Figure S2).

### Mediation Analysis

**Figure 4:**
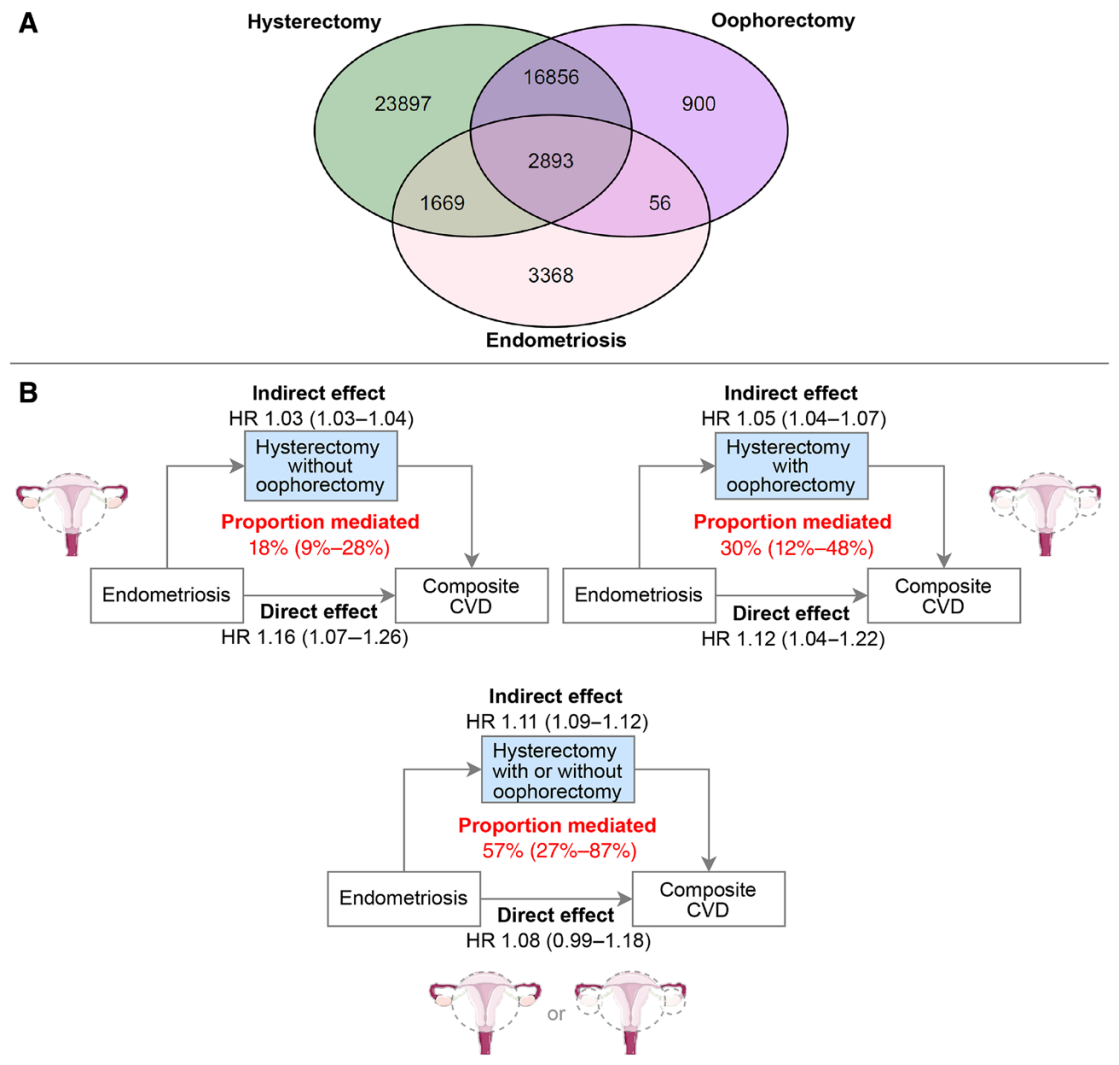
Mediation analysis. Mediation analysis of the association between endometriosis and composite CVD using a counterfactual framework. In **A**, distribution of women with hysterectomy, oophorectomy, and/or endometriosis is shown in a Venn diagram. In **B**, the total effect of endometriosis on cardiovascular disease risk was decomposed into the direct effect and indirect effect via (1) hysterectomy without oophorectomy (n=25 566), (2) hysterectomy with oophorectomy (n=19 749), and (3) hysterectomy with or without oophorectomy (n = 45 315). HRs and 95% CIs were estimated using weighted Cox proportional hazards models. Stabilized weights were derived from logistic regression models predicting the mediator. The proportion mediated was calculated as the log ratio of the indirect to the total effect. All estimates are reported with their respective 95% CIs between brackets. Hysterectomy and oophorectomy counts are based on baseline data including missing values and the analysis is performed on imputed data. CVD, cardiovascular disease; and HR, hazard ratio. Image provided by Servier Medical Art (https://smart.servier.com/), licensed under CC BY 4.0 (https://creativecommons.org/licenses/by/4.0/).

The results of the mediation analyses can be found in Figure 4. The median latency between endometriosis and hysterectomy was 1 year (25th‐75th percentile 0–3), and between hysterectomy and composite CVD it was 26.6 years (25th‐75th percentile 21.8–34.8). Mediation analysis suggested that hysterectomy without oophorectomy may mediate the relationship between endometriosis and composite CVD, with an indirect HR of 1.03 (95% CI 1.03–1.04, *P*<0.001) and a proportion mediated of 18% (95% CI 9%–28%). Hysterectomy with oophorectomy appeared to show a stronger mediating effect, with an indirect HR of 1.05 (95% CI 1.03–1.07, *P*<0.001) and a proportion mediated of 30% (95% CI 12%–49%). The strongest mediation signal was observed for hysterectomy with or without oophorectomy, with an indirect HR of 1.11 (95% CI 1.09–1.12, *P*<0.001), corresponding to a proportion mediated of 57% (95% CI 27%–87%).

### Sensitivity Analysis

The results of the sensitivity analyses are shown in Figures S3–S5 and Table S2. Stepwise adjustment for covariates did not materially alter the association between endometriosis and composite CVD (Figure S3), indicating robustness to confounder control. The association was also consistent for non–self‐reported endometriosis, for cases diagnosed before hysterectomy, when excluding coexisting adenomyosis, when using time‐to‐event instead of age as the time scale, and in analyses censoring events 3 years after endometriosis diagnosis as well as in Fine–Gray competing risks models accounting for non‐CVD mortality (Figure S4). Complete case analysis yielded similar findings (Figure S5). For the mediation analysis, restricting to endometriosis cases diagnosed before hysterectomy resulted in very similar results, and classical mediation analysis also produced comparable findings (Table S2).

In mediation analyses stratified by HRT use, no mediation could be evaluated in the ever HRT use subgroup because the total effect between endometriosis and CVD in the counterfactual approach model was not statistically significant. In contrast, among women who had never used HRT, hysterectomy with or without oophorectomy mediated 37% (95% CI 10%–61%) of the total association (indirect HR 1.09 [95% CI 1.07–1.11], *P*<0.001; direct HR 1.17 [95% CI 1.02–1.34]). When considered separately, both hysterectomy with oophorectomy (13% mediation [95% CI 1%–25%]; indirect HR 1.03 [95% CI 1.01–1.05], *P*<0.001; direct HR 1.23 [95% CI 1.08–1.41]) and hysterectomy without oophorectomy (25% mediation [CI 6%–43%]; indirect HR 1.06 [95% CI 1.04–1.07], *P*<0.001; direct HR 1.18 [95% CI 1.04–1.36]) showed significant mediation effects.

In our sensitivity analysis on the association between hysterectomy and CVD, hysterectomy was associated with a higher risk of composite CVD (HR 1.24 [95% CI 1.21–1.28], *P*<0.001) in women without endometriosis and in women with endometriosis (HR 1.22 [95% CI 1.02–1.45], *P*=0.034). This link remained when looking at hysterectomy without oophorectomy in women without endometriosis (HR 1.26 [95% CI 1.21–1.31], *P*<0.001) but disappeared in women with endometriosis (HR 1.02 [95% CI 0.85–1.22], *P*=0.83).

## DISCUSSION

In this large cohort of 264 740 women free of CVD at baseline, we found that endometriosis was associated with an 18% higher risk of incident CVD. This higher risk was mainly driven by CHD. Conversely, women with endometriosis had a 12% lower risk of all‐cause mortality. Mediation analysis suggests that hysterectomy mediates the association between endometriosis and CVD, with an estimated 56% of the increased cardiovascular risk potentially explained through this pathway.

### Comparison with Existing Literature

Our findings indicate that endometriosis is associated with a higher risk of CHD, and composite CVD, consistent with previous meta‐analyses.^5^, ^6^, ^7^, ^8^ The random‐effects meta‐analysis by Saad *et al*.^7^ included seven studies with a total of 1 407 875 participants identified through a systematic literature search conducted up to November 2024. They reported a HR of 1.13 (95% CI 1.03–1.25) for composite CVD and an HR of 1.28 (95% CI 1.17–1.40) for ischemic heart disease, which is similar to our definition of CHD.

In contrast, we did not observe statistically significant associations with stroke, MI, PAD, or cardiovascular mortality, partially diverging from earlier findings. Previous meta‐analyses reported endometriosis being related to a higher risk of stroke.^5^, ^6^, ^7^, ^8^ For instance, Saad et al.^7^ reported a HR of 1.11 (95% CI 1.04–1.18). Similarly, Okoli et al.^5^ reported a significant increased risk of PAD (OR 1.55; 95% CI 1.35–1.78). Notably, only Winata *et al*.^8^ examined MI separately and reported a significantly increased risk (OR 1.53 [95% CI 1.18–1.98]), which we did not observe in our cohort. One potential explanation for this discrepancy lies in differences in participant demographics. Prior meta‐analyses primarily included studies with relatively young populations, with mean baseline ages often below 40 years. In contrast, the median age at baseline in our cohort was 57 years. In an older cohort, a high baseline risk of disease, along with survivor bias (ie, where only the healthier women with endometriosis remain) may together mask any additional risk attributable to endometriosis. However, our subgroup analysis by age at baseline for composite CVD did not yield any significantly different results.

We also observed a significant protective association between endometriosis and all‐cause mortality. This finding aligns with trends reported in previous meta‐analyses, although those did not reach statistical significance (HR 0.79 [95% CI 0.56–1.10]^7^ and OR 0.88 [95% CI 0.72–1.08]^8^). These 2 meta‐analyses included 2^14^, ^25^ and 4^14^, ^26^, ^27^, ^28^ studies for their analysis of all‐cause mortality, each adding up to 582 299 and 1 003 322 participants, respectively. Only 1 previous study examined cardiovascular mortality in relation to endometriosis and reported a decreased risk (HR 0.55 [95% CI 0.47–0.65]).^28^ Although our findings for cardiovascular mortality were not statistically significant, the result was directionally similar (HR 0.86 [95% CI 0.64–1.15]).

### CVD Risk and Mortality Paradox

The apparent paradox of an increased risk of composite CVD but a reduced risk of both CVD‐specific and all‐cause mortality may be largely explained by detection and surveillance bias. Individuals with endometriosis may experience higher levels of health care contact and ongoing medical surveillance following diagnosis, which could increase the likelihood of earlier detection of cardiometabolic risk factors, such as impaired glucose tolerance or hypertension, as well as earlier diagnosis of non‐fatal cardiovascular outcomes such as angina. The diagnosis of endometriosis typically occurs at a relatively young age, when clinical CVD is uncommon, but when long‐term risk factor accumulation may still be modifiable under increased medical attention.

Treatments for endometriosis may partly explain the observed inverse association with all‐cause mortality. Several endometriosis treatments have been associated with reduced cancer risk, which could influence long‐term mortality outcomes. For example, surgical interventions such as oophorectomy and excision of visible endometriosis have been linked to a lower risk of ovarian cancer,^29^ while hormonal therapies have been associated with reduced risks of liver, thyroid, breast, and lung cancer.^30^

Finally, residual selection bias may also play a role, as UK Biobank participants tend to be healthier and of higher socioeconomic status than the general population, potentially limiting the generalizability of these findings.^31^ In addition, if women classified as having endometriosis were required to survive until diagnosis or ascertainment of the condition, some degree of immortal time bias may have been introduced, potentially contributing to the observed inverse association with mortality. Further research is needed to disentangle these potential mechanisms.

### Is the Relationship Between Endometriosis and CVD Risk Causal?

Causal inference is limited by the observational design of both our study and the previously conducted meta‐analyses. However, recent Mendelian randomization (MR) studies provide evidence for both a potential causal effect of endometriosis on CVD and shared underlying mechanisms. One study reported a causal association between endometriosis and stroke, suggesting that endometriosis may directly increase CVD risk.^32^ Other MR findings, however, point to common biological pathways rather than direct causality. Genetic predisposition to altered coagulation factors (eg, ADAMTS13, vWF),^33^ vascular signaling proteins (eg, EPHB4),^34^ and elevated triglycerides^35^ increase not only the risk of endometriosis, but also the risk of CVD. These findings support the leading hypotheses that systemic inflammation, accelerated atherogenesis, and metabolic dysregulation are underlying the relationship between endometriosis and CVD.^9^, ^11^, ^12^, ^13^ Overall, these MR results highlight a complex interplay between endometriosis and CVD‐related traits, involving both potential causal effects and common etiological pathways. However, our findings suggest that conventional CVD risk factors such as low‐density lipoprotein cholesterol, systolic blood pressure, and lipoprotein(a) only partially, if at all, account for the association between endometriosis and CVD, as the effect estimates did not change after adjustment. Ultimately, our results cannot determine whether the observed association between endometriosis and CVD is attributable to a true causal relationship, or whether it reflects detection bias where individuals with endometriosis are more likely to seek medical care and thus have earlier diagnoses.

### Endometriosis Treatment as Potential Mediators

Another possible explanation for the observed association between endometriosis and CVD is mediation through treatment exposure. Management of endometriosis often involves a combination of pharmacological and surgical interventions. Medical management involves analgesics like non‐steroidal anti‐inflammatory drugs, and hormonal therapies (such as combined oral contraceptives, progestins, and gonadotropin‐releasing hormone analogs), while surgical options include resection of peritoneal and/or deep infiltrating lesions, ovarian cystectomy, or in advanced cases hysterectomy and/or oophorectomy.^36^, ^37^

In our mediation analysis, hysterectomy with or without oophorectomy showed a substantial mediating effect (proportion mediated: 56% [95% CI 27%–85%]; indirect effect HR 1.11 [95% CI 1.09–1.12]). The effect remained significant but was smaller when considering hysterectomy without oophorectomy. These results need to be interpreted with caution, since hysterectomy may serve as a proxy for more severe endometriosis rather than acting as a true mediator. Unfortunately, there ar no data on endometriosis disease stage, burden, pain, or effects on fertility beyond the stillbirth or miscarriage variables in the UKB, so we cannot rule out that hysterectomy is serving as a proxy for these factors. However, both hysterectomy and oophorectomy have previously been identified as risk factors for CVD,^16^, ^38^, ^39^, ^40^ and partial mediators of the association between endometriosis and CVD.^14^, ^15^, ^16^ The hysterectomy/oophorectomy‐CVD link appears stronger among women who undergo surgery at a younger age,^16^, ^38^, ^39^, ^40^ possibly attributable to an earlier decline in estrogen levels and subsequent earlier onset of menopause. This link between hysterectomy and CVD is independent of HRT use.^41^, ^42^ Increasing evidence suggests a link between earlier menopause and elevated CVD risk, although the causal nature of this relationship remains uncertain.^13^, ^38^, ^43^, ^44^, ^45^ Conversely, increased CVD risk has also been observed in women who had a hysterectomy with ovarian conservation,^16^, ^38^, ^39^, ^41^ challenging the decline in estrogen levels hypothesis. However, several studies have shown that even when the ovaries are conserved, hysterectomy is still associated with an earlier onset of menopause.^46^, ^47^, ^48^ In our sensitivity analysis, there was generally a strong association between hysterectomy and CVD, except in the subgroup of women with endometriosis who underwent hysterectomy without oophorectomy, where the association was attenuated. It is important to note that evidence linking hysterectomy and oophorectomy to CVD risk stems almost exclusively from observational studies. Only 1 randomized controlled trial has found that hysterectomy was related to increased levels of serum inflammatory markers.^49^ Consequently, it is not possible to conclude that hysterectomy and oophorectomy themselves are direct causes of CVD. Future studies are needed to investigate this causal pathway in greater depth.

Medical management options are also, to varying degrees, linked to an increased risk of CVD. The use of non‐steroidal anti‐inflammatory drugs has been associated with a higher risk of heart failure, hypertension, and thrombotic events.^50^ Opioids have been linked to adverse cardiovascular outcomes, including acute coronary syndrome and endocarditis.^51^ The relationship between estrogen/progestin therapy and CVD is complex and findings remain inconsistent. While some cardiovascular risk factors may improve with estrogen/progestin therapy,^52^, ^53^ evidence also points to increased risks of hypertension and CHD.^54^, ^55^ For gonadotropin‐releasing hormone analogs, the picture is also nuanced, with studies suggesting an increased risk of hypertension in women, but a decreased risk of ischemic heart disease.^56^ In our subgroup analysis, we found no significant interaction between ever use of OCP or HRT and endometriosis.

### Strengths and Limitations

A major strength of this study is the use of the UKB, a large, deeply phenotyped cohort with long‐term follow‐up and access to linked health records. Its prospective design reduces the risk of recall bias. In addition, we adjusted our analyses for a wide range of potential confounders, including socioeconomic and lifestyle factors such as the Townsend Deprivation Index, ethnicity, and the International Physical Activity Questionnaire physical activity score, as well as emerging cardiovascular risk factors like lipoprotein(a) and female‐specific risk factors like age at menarche and OCP use, but residual confounding cannot be fully excluded because of the observational nature of the study. Another strength is the application of mediation analysis, which enabled exploring potential mechanisms linking endometriosis and CVD, although the potential to draw causal conclusions remains limited.

Several limitations should be acknowledged. Misclassification of endometriosis is possible because of reliance on clinical and self‐reported diagnoses, with asymptomatic or undiagnosed cases potentially missed. However, sensitivity analyses comparing self‐reported and clinically confirmed cases yielded similar results, supporting the robustness of our findings. All outcomes were based on registry data and not collected systematically, which may introduce measurement bias. However, in a sensitivity analysis excluding cases where endometriosis was diagnosed in the same year or after hysterectomy, the estimates remained unchanged, suggesting the results are at least partially robust to this potential bias. Moreover, we performed another sensitivity analysis to assess temporal ordering of exposure, mediator, and outcome. Again, the results were similar to the findings of the primary analysis. The median time from diagnosis of endometriosis to hysterectomy was substantially shorter than the median time from hysterectomy to CVD, consistent with hysterectomy being a proximal response to endometriosis. Furthermore, the hysterectomy variable in the UKB lacks specificity about the type of procedure performed. In addition, because of the observational design of UKB, we cannot draw any definite conclusions about causal relationships between endometriosis and CVD. However, to overcome confounding to some extent, we adjusted for conventional and emerging cardiovascular risk factors, lifestyle and demographic variables, as well as female‐specific factors. We decided to adjust for cardiovascular risk factors because these risk factors and endometriosis share common antecedents and biological pathways. Higher body mass index is linked with increased risk of endometriosis,^57^ and women with endometriosis show higher incidence of hypertension and dyslipidemia.^10^ Moreover, lifestyle and demographic variables have been shown to be associated with an increased incidence of endometriosis, including ethnicity, alcohol use, and cigarette smoking.^58^ Additionally, we also adjusted for OCP intake and age at menarche as they are a common risk factor for both endometriosis and CVD.^59^, ^60^, ^61^, ^62^ However, in our sensitivity analysis, results were similar across all levels of adjustments. To support confounder selection, we constructed a directed acyclic graph (Figure S6). While we examined subgroups based on polycystic ovary syndrome and history of stillbirth, the limited number of cases (n=981 and n=6587, respectively) may have reduced power to detect significant interaction. This is particularly relevant for the stillbirth subgroup, where the point estimate differed from the primary analysis, and variation in the association between endometriosis and CVD cannot be ruled out. Another limitation is that our data on HRT use are not detailed enough to distinguish between initiation near menopause versus later initiation, which has been associated with cardioprotective effects.^63^ Many participants were postmenopausal at baseline, limiting generalizability to younger women; however, as CVD primarily affects older populations, their inclusion is appropriate for this outcome.

### Implications for Research and Practice

Our study adds to the growing body of evidence linking endometriosis to CVD, and provides new insights into potential mechanisms, including the possible mediating role of hysterectomy and oophorectomy. Although the association between endometriosis and CVD appears modest in relative terms, it may still be relevant from a public health perspective given the prevalence of endometriosis and the substantial baseline risk of CVD. Because both our study and most existing research are observational in nature, we cannot draw firm causal conclusions from our mediation analysis. Future research should, therefore, focus on disentangling the biological and treatment‐related mechanisms underlying this association. Long‐term prospective studies, complemented by mechanistic and interventional research, are needed to clarify whether the link between endometriosis and CVD is driven by hormonal changes, inflammation, shared risk factors, or treatment effects. Collecting more granular data on disease severity and medical management, including hormonal therapies and analgesic use, would also help reduce confounding by indication and strengthen causal inference.

## CONCLUSIONS

In this large‐scale study, endometriosis was associated with an increased risk of CVD, but all‐cause mortality was lower among affected women. Hysterectomy appeared to mediate parts of the observed association between endometriosis and CVD. Whether hysterectomy may proxy endometriosis severity instead of being a true mediator remains to be elucidated. These findings emphasize the need for in‐depth research to unravel the causal mechanisms linking endometriosis, its treatments, and cardiovascular outcomes. A clearer understanding of these pathways may eventually inform clinical practice, potentially leading to refined treatment strategies and targeted cardiovascular risk assessment in women with endometriosis.

## Sources of Funding

This research was funded in whole or in part by the Austrian Science Fund (FWF) [10.55776/T1253].

## Disclosures

P.W. reports consultancy fees from Novartis Pharmaceuticals outside the submitted work and consultancy fees as statistical reviewer for the Lancet group. The remaining authors have no disclosures to report.

## Supporting information

Tables S1‐S2
Figures S1‐S6
References [^64^, ^65^, ^66^]

## References

[1] Roth GA , Mensah GA , Johnson CO , Addolorato G , Ammirati E , Baddour LM , Barengo NC , Beaton AZ , Benjamin EJ , Benziger CP , et al. Global burden of cardiovascular diseases and risk factors, 1990‐2019: update from the GBD 2019 study. J Am Coll Cardiol. 2020;76:2982–3021. doi: 10.1016/j.jacc.2020.11.010 33309175 PMC7755038

[2] Leening MJG , Ferket BS , Steyerberg EW , Kavousi M , Deckers JW , Nieboer D , Heeringa J , Portegies MLP , Hofman A , Ikram MA , et al. Sex differences in lifetime risk and first manifestation of cardiovascular disease: prospective population based cohort study. BMJ. 2014;349:g5992. doi: 10.1136/bmj.g5992 25403476 PMC4233917

[3] Nguyen AH , Hurwitz M , Sullivan SA , Saad A , Kennedy JLW , Sharma G . Update on sex specific risk factors in cardiovascular disease. Front Cardiovasc Med. 2024;11:1352675. doi: 10.3389/fcvm.2024.1352675 38380176 PMC10876862

[4] Taylor HS , Kotlyar AM , Flores VA . Endometriosis is a chronic systemic disease: clinical challenges and novel innovations. Lancet. 2021;397:839–852. doi: 10.1016/S0140-6736(21)00389-5 33640070

[5] Okoli U , Charoenngam N , Ponvilawan B , Jaroenlapnopparat A , Mettler S , Obiejesie O . Endometriosis and risk of cardiovascular disease: systematic review and meta‐analysis. J Womens Health (Larchmt). 2023;32:1328–1339. doi: 10.1089/jwh.2023.0091 37856152

[6] Poeta do Couto C , Policiano C , Pinto FJ , Brito D , Caldeira D . Endometriosis and cardiovascular disease: a systematic review and meta‐analysis. Maturitas. 2023;171:45–52. doi: 10.1016/j.maturitas.2023.04.001 37075537

[7] Saad M , Ansari I , Ibrahim ZS , Batool RM , Ahsan SI , Arshad MS , Collins P , Ahmed R . Increased risk of cardiovascular disease in women with endometriosis: a systematic review and meta‐analysis. Eur J Obstet Gynecol Reprod Biol. 2025;312:114081. doi: 10.1016/j.ejogrb.2025.114081 40450807

[8] Winata IG , Immanuel SS , Leonardo L , Rinaldi FX , Tandecxi G , Wijaya R . Examining the interplay between endometriosis and later‐life cerebro‐cardiovascular diseases: a systematic review, meta‐analysis, and trial sequential analysis. Narra J. 2025;5:e1935. doi: 10.52225/narra.v5i1.1935 40352216 PMC12059851

[9] Tan J , Taskin O , Iews M , Lee AJ , Kan A , Rowe T , Bedaiwy MA . Atherosclerotic cardiovascular disease in women with endometriosis: a systematic review of risk factors and prospects for early surveillance. Reprod Biomed Online. 2019;39:1007–1016. doi: 10.1016/j.rbmo.2019.05.021 31735549

[10] Mu F , Rich‐Edwards J , Rimm EB , Spiegelman D , Forman JP , Missmer SA . Association between endometriosis and hypercholesterolemia or hypertension. Hypertension. 2017;70:59–65. doi: 10.1161/HYPERTENSIONAHA.117.09056 28559401 PMC6469492

[11] Vazgiourakis VM , Zervou MI , Papageorgiou L , Chaniotis D , Spandidos DA , Vlachakis D , Eliopoulos E , Goulielmos GN . Association of endometriosis with cardiovascular disease: genetic aspects (review). Int J Mol Med. 2023;51:29. doi: 10.3892/ijmm.2023.5232 36799179 PMC9943539

[12] Marchandot B , Curtiaud A , Matsushita K , Trimaille A , Host A , Faller E , Garbin O , Akladios C , Jesel L , Morel O . Endometriosis and cardiovascular disease. Eur Heart J Open. 2022;2:oeac001. doi: 10.1093/ehjopen/oeac001 35919664 PMC9242051

[13] Taskin O , Rikhraj K , Tan J , Sedlak T , Rowe TC , Bedaiwy MA . Link between endometriosis, atherosclerotic cardiovascular disease, and the health of women midlife. J Minim Invasive Gynecol. 2019;26:781–784. doi: 10.1016/j.jmig.2019.02.022 31028947

[14] Okoth K , Wang J , Zemedikun D , Thomas GN , Nirantharakumar K , Adderley NJ . Risk of cardiovascular outcomes among women with endometriosis in the United Kingdom: a retrospective matched cohort study. BJOG. 2021;128:1598–1609. doi: 10.1111/1471-0528.16692 33683770

[15] Mu F , Rich‐Edwards J , Rimm EB , Spiegelman D , Missmer SA . Endometriosis and risk of coronary heart disease. Circ Cardiovasc Qual Outcomes. 2016;9:257–264. doi: 10.1161/CIRCOUTCOMES.115.002224 27025928 PMC4940126

[16] Farland LV , Degnan WJ , Bell ML , Kasner SE , Liberman AL , Shah DK , Rexrode KM , Missmer SA . Laparoscopically confirmed endometriosis and risk of incident stroke: a prospective cohort study. Stroke. 2022;53:3116–3122. doi: 10.1161/STROKEAHA.122.039250 35861076 PMC9529799

[17] Sudlow C , Gallacher J , Allen N , Beral V , Burton P , Danesh J , Downey P , Elliott P , Green J , Landray M , et al. UK biobank: an open access resource for identifying the causes of a wide range of complex diseases of middle and old age. PLoS Med. 2015;12:e1001779. doi: 10.1371/journal.pmed.1001779 25826379 PMC4380465

[18] Allen N , Sudlow C , Downey P , Peakman T , Danesh J , Elliott P , Gallacher J , Green J , Matthews P , Pell J , et al. UK biobank: current status and what it means for epidemiology. Health Policy Technol. 2012;1:123–126. doi: 10.1016/j.hlpt.2012.07.003

[19] UK Biobank . First Occurrence of Health Outcomes Defined by 3‐character ICD10 code . 2019. Accessed May 27, 2025. https://biobank.ndph.ox.ac.uk/ukb/ukb/docs/first_occurrences_outcomes.pdf. Updated September 2019.

[20] R Core Team . R: A language and environment for statistical computing. 2013.

[21] VanderWeele TJ . Causal mediation analysis with survival data. Epidemiology. 2011;22:582–585. doi: 10.1097/EDE.0b013e31821db37e 21642779 PMC3109321

[22] Holst KK , Scheike TH , Hjelmborg JB . The liability threshold model for censored twin data. Comput Stat Data Anal. 2016;93:324–335. doi: 10.1016/j.csda.2015.01.014

[23] Baron RM , Kenny DA . The moderator‐mediator variable distinction in social psychological research: conceptual, strategic, and statistical considerations. J Pers Soc Psychol. 1986;51:1173–1182. doi: 10.1037/0022-3514.51.6.1173 3806354

[24] Fine JP , Gray RJ . A proportional hazards model for the subdistribution of a competing risk. J Am Stat Assoc. 1999;94:496–509. doi: 10.1080/01621459.1999.10474144

[25] Havers‐Borgersen E , Hartwell D , Ekelund C , Butt JH , Østergaard L , Holgersson C , Schou M , Køber L , Fosbøl EL . Endometriosis and long‐term cardiovascular risk: a nationwide Danish study. Eur Heart J. 2024;45:4734–4743. doi: 10.1093/eurheartj/ehae563 39219447

[26] Blom JN , Velez MP , McClintock C , Shellenberger J , Pudwell J , Brogly SB , Bougie O . Endometriosis and cardiovascular disease: a population‐based cohort study. CMAJ Open. 2023;11:E227–E236. doi: 10.9778/cmajo.20220144 PMC10000901 36882211

[27] Chiang H‐J , Lan K‐C , Yang Y‐H , Chiang JY , Kung F‐T , Huang F‐J , Lin Y‐J , Su Y‐T , Sung P‐H . Risk of major adverse cardiovascular and cerebrovascular events in Taiwanese women with endometriosis. J Formos Med Assoc. 2021;120:327–336. doi: 10.1016/j.jfma.2020.10.005 33268157

[28] Saavalainen L , But A , Tiitinen A , Härkki P , Gissler M , Haukka J , Heikinheimo O . Mortality of midlife women with surgically verified endometriosis—a cohort study including 2.5 million person‐years of observation. Hum Reprod. 2019;34:1576–1586. doi: 10.1093/humrep/dez074 31265075

[29] Melin A‐S , Lundholm C , Malki N , Swahn M‐L , Sparèn P , Bergqvist A . Hormonal and surgical treatments for endometriosis and risk of epithelial ovarian cancer. Acta Obstet Gynecol Scand. 2013;92:546–554. doi: 10.1111/aogs.12123 23560387

[30] Lee HJ , Lee B , Choi H , Lee M , Lee K , Lee TK , Hwang SO , Kim YB . Hormone replacement therapy and risks of various cancers in postmenopausal women with de novo or a history of endometriosis. Cancers (Basel). 2024;16:809. doi: 10.3390/cancers16040809 38398200 PMC10886569

[31] Fry A , Littlejohns TJ , Sudlow C , Doherty N , Adamska L , Sprosen T , Collins R , Allen NE . Comparison of sociodemographic and health‐related characteristics of UK biobank participants with those of the general population. Am J Epidemiol. 2017;186:1026–1034. doi: 10.1093/aje/kwx246 28641372 PMC5860371

[32] Zheng M , Zheng S . Endometriosis increases the risk of stroke: a mendelian randomization study. Stroke. 2023;54:e30–e33. doi: 10.1161/STROKEAHA.122.041163 36689595

[33] Li Y , Liu H , Ye S , Zhang B , Li X , Yuan J , Du Y , Wang J , Yang Y . The effects of coagulation factors on the risk of endometriosis: a mendelian randomization study. BMC Med. 2023;21:195. doi: 10.1186/s12916-023-02881-z 37226166 PMC10210381

[34] Ling S , Xie D , Huang L , Huang S , Tian C , Huang L , Chen R , Qin L , Qin X . Identification of EPHB4 as a potential causal gene and therapeutic target for endometriosis using mendelian randomization. Hereditas. 2025;162:92. doi: 10.1186/s41065-025-00457-w 40450304 PMC12126875

[35] Zhang H , Fan Y , Li H , Feng X , Yue D . Genetic association of serum lipids and lipid‐modifying targets with endometriosis: trans‐ethnic mendelian‐randomization and mediation analysis. PLoS One. 2024;19:e0301752. doi: 10.1371/journal.pone.0301752 38820493 PMC11142702

[36] Ferrero S , Evangelisti G , Barra F . Current and emerging treatment options for endometriosis. Expert Opin Pharmacother. 2018;19:1109–1125. doi: 10.1080/14656566.2018.1494154 29975553

[37] Horne AW , Missmer SA . Pathophysiology, diagnosis, and management of endometriosis. BMJ. 2022;379:e070750. doi: 10.1136/bmj-2022-070750 36375827

[38] Wang Z , Li X , Zhang D . Impact of hysterectomy on cardiovascular disease and different subtypes: a meta‐analysis. Arch Gynecol Obstet. 2022;305:1255–1263. doi: 10.1007/s00404-021-06240-2 34524503

[39] Chen Y , Li F , Liang L , Hua H , Liu S , Yu Z , Chen Q , Huang S , Qin P . Examining the association of hysterectomy with and without oophorectomy on cardiovascular disease and all‐cause, cardiovascular or cancer mortality: a systematic review and meta‐analysis. BJOG. 2024;131:1444–1455. doi: 10.1111/1471-0528.17843 38747094

[40] Thao V , Borah B , Stewart EA , Farland LV , Laughlin‐Tommaso SK , Khan Z , Coutinho TD , Rassier SLC . Cardiovascular disease after hysterectomy in the nurses' health study and nurses' health study II. Obstet Gynecol. 2025;146:85–93. doi: 10.1097/AOG.0000000000005902 40341551 PMC12221304

[41] Ingelsson E , Lundholm C , Johansson ALV , Altman D . Hysterectomy and risk of cardiovascular disease: a population‐based cohort study. Eur Heart J. 2011;32:745–750. doi: 10.1093/eurheartj/ehq477 21186237

[42] Farland LV , Rice MS , Degnan WJ , Rexrode KM , Manson JE , Rimm EB , Rich‐Edwards J , Stewart EA , Cohen Rassier SL , Robinson WR , et al. Hysterectomy with and without oophorectomy, tubal ligation, and risk of cardiovascular disease in the nurses' health study II. J Womens Health (Larchmt). 2023;32:747–756. doi: 10.1089/jwh.2022.0207 37155739 PMC10354306

[43] Younis JS , Taylor HS . Is there an association between endometriosis, early menopause, and cardiovascular disease? J Clin Endocrinol Metab. 2024;109:dgae508. doi: 10.1210/clinem/dgae508 39083665

[44] Gavin KM , Jankowski C , Kohrt WM , Stauffer BL , Seals DR , Moreau KL . Hysterectomy is associated with large artery stiffening in estrogen‐deficient postmenopausal women. Menopause. 2012;19:1000–1007. doi: 10.1097/gme.0b013e31825040f9 22692329 PMC3428437

[45] Tschiderer L , Peters SAE , van der Schouw YT , van Westing AC , Tong TYN , Willeit P , Seekircher L , Moreno‐Iribas C , Huerta JM , Crous‐Bou M , et al. Age at menopause and the risk of stroke: observational and mendelian randomization analysis in 204 244 postmenopausal women. J Am Heart Assoc. 2023;12:e030280. doi: 10.1161/JAHA.123.030280 37681566 PMC10547274

[46] Farquhar CM , Sadler L , Harvey SA , Stewart AW . The association of hysterectomy and menopause: a prospective cohort study. BJOG. 2005;112:956–962. doi: 10.1111/j.1471-0528.2005.00696.x 15957999

[47] Moorman PG , Myers ER , Schildkraut JM , Iversen ES , Wang F , Warren N . Effect of hysterectomy with ovarian preservation on ovarian function. Obstet Gynecol. 2011;118:1271–1279. doi: 10.1097/AOG.0b013e318236fd12 22067716 PMC3223258

[48] Ahn EH , Bai SW , Song CH , Kim JY , Jeong KA , Kim SK , Lee JS , Kwon JY , Park KH . Effect of hysterectomy on conserved ovarian function. Yonsei Med J. 2002;43:53–58. doi: 10.3349/ymj.2002.43.1.53 11854933

[49] Heliövaara‐Peippo S , Oksjoki R , Halmesmäki K , Kaaja R , Teperi J , Grenman S , Kivelä A , Surcel H‐M , Tomas E , Tuppurainen M , et al. The effect of hysterectomy or levonorgestrel‐releasing intrauterine system on cardiovascular disease risk factors in menorrhagia patients: a 10‐year follow‐up of a randomised trial. Maturitas. 2011;69:354–358. doi: 10.1016/j.maturitas.2011.05.004 21684096

[50] Schjerning A‐M , McGettigan P , Gislason G . Cardiovascular effects and safety of (non‐aspirin) NSAIDs. Nat Rev Cardiol. 2020;17:574–584. doi: 10.1038/s41569-020-0366-z 32322101

[51] Krantz MJ , Palmer RB , Haigney MCP . Cardiovascular complications of opioid use: JACC state‐of‐the‐art review. J Am Coll Cardiol. 2021;77:205–223. doi: 10.1016/j.jacc.2020.11.002 33446314

[52] Nudy M , Aragaki AK , Jiang X , Manson JE , Shadyab AH , Jung SY , Martin LW , Wild RA , Womack C , Mouton CP , et al. Long‐term changes to cardiovascular biomarkers after hormone therapy in the Women's Health Initiative hormone therapy clinical trials. Obstet Gynecol. 2025;145:357–367. doi: 10.1097/AOG.0000000000005862 40014858 PMC11972549

[53] Vaisar T , Gordon JL , Wimberger J , Heinecke JW , Hinderliter AL , Rubinow DR , Girdler SS , Rubinow KB . Perimenopausal transdermal estradiol replacement reduces serum HDL cholesterol efflux capacity but improves cardiovascular risk factors. J Clin Lipidol. 2021;15:151–161.e0. doi: 10.1016/j.jacl.2020.11.009 33288437 PMC7887026

[54] Madika A‐L , MacDonald CJ , Fournier A , Mounier‐Vehier C , Béraud G , Boutron‐Ruault M‐C . Menopausal hormone therapy and risk of incident hypertension: role of the route of estrogen administration and progestogens in the E3N cohort. Menopause. 2021;28:1204–1208. doi: 10.1097/GME.0000000000001839 34581294

[55] Manson JE , Hsia J , Johnson KC , Rossouw JE , Assaf AR , Lasser NL , Trevisan M , Black HR , Heckbert SR , Detrano R , et al. Estrogen plus progestin and the risk of coronary heart disease. N Engl J Med. 2003;349:523–534. doi: 10.1056/NEJMoa030808 12904517

[56] Lim MWS , Lucas‐Herald AK , Mason A , Delles C , Connelly PJ . Sex differences in the cardiovascular effects of GnRH analogues. J Endocrinol. 2024;261:e230309. doi: 10.1530/JOE-23-0309 38265843

[57] Liao L , Pan Z , Li Y . Endometriosis as a risk factor: impact on IVF outcomes and reproductive parameters: a systematic review and meta‐analysis. Arch Gynecol Obstet. 2025;312:1085–1093. doi: 10.1007/s00404-025-08137-w 40748500 PMC12414075

[58] Missmer SA , Hankinson SE , Spiegelman D , Barbieri RL , Marshall LM , Hunter DJ . Incidence of laparoscopically confirmed endometriosis by demographic, anthropometric, and lifestyle factors. Am J Epidemiol. 2004;160:784–796. doi: 10.1093/aje/kwh275 15466501

[59] Vercellini P , Bandini V , Viganò P , Di Stefano G , Merli CEM , Somigliana E . Proposal for targeted, neo‐evolutionary‐oriented, secondary prevention of early‐onset endometriosis and adenomyosis. Part I: pathogenic aspects. Hum Reprod. 2024;39:1–17. doi: 10.1093/humrep/dead229 37951243 PMC10876119

[60] Vercellini P , Bandini V , Viganò P , Ambruoso D , Cetera GE , Somigliana E . Proposal for targeted, neo‐evolutionary‐oriented secondary prevention of early‐onset endometriosis and adenomyosis. Part II: medical interventions. Hum Reprod. 2024;39:18–34. doi: 10.1093/humrep/dead206 37951241 PMC11639102

[61] Okoth K , Chandan JS , Marshall T , Thangaratinam S , Thomas GN , Nirantharakumar K , Adderley NJ . Association between the reproductive health of young women and cardiovascular disease in later life: umbrella review. BMJ. 2020;371:m3502. doi: 10.1136/bmj.m3502 33028606 PMC7537472

[62] Lu M‐Y , Niu J‐L , Liu B . The risk of endometriosis by early menarche is recently increased: a meta‐analysis of literature published from 2000 to 2020. Arch Gynecol Obstet. 2023;307:59–69. doi: 10.1007/s00404-022-06541-0 35377041

[63] Hodis HN , Mack WJ . Menopausal hormone replacement therapy and reduction of all‐cause mortality and cardiovascular disease: it is about time and timing. Cancer J. 2022;28:208–223. doi: 10.1097/PPO.0000000000000591 35594469 PMC9178928

[64] van Buuren S , Groothuis‐Oudshoorn K , Robitzsch A , Vink G , Doove L , Jolani S , et al. Package ‘mice’. Computer software. 2015.

[65] Burgess S , White IR , Resche‐Rigon M , Wood AM . Combining multiple imputation and meta‐analysis with individual participant data. Stat Med. 2013;32:4499–4514. doi: 10.1002/sim.5844 23703895 PMC3963448

[66] Textor J , van der Zander B , Gilthorpe MS , Liskiewicz M , Ellison GT . Robust causal inference using directed acyclic graphs: the R package ‘dagitty’. Int J Epidemiol. 2016;45:1887–1894. doi: 10.1093/ije/dyw341 28089956

