# Supplementary material for: Association Between Endometriosis and Cardiovascular Disease: Insights From the UK Biobank
Source: J Am Heart Assoc. 2026 Jul 10;15(14):e048454. doi: 10.1161/JAHA.125.048454 (PMC13477338; doi:10.1161/JAHA.125.048454)
Supplement: Supplementary file 1 [file JAH3-15-e048454-s001.pdf]

# Supplementary methods

## Definition of covariates

At recruitment, participants' weight and height were measured, and body mass index (BMI) ($kg/m^2$) was calculated. Systolic and diastolic blood pressure (mmHg) were calculated using the mean of two readings with an automated device, at least one minute apart. Low-density lipoprotein (LDL)-cholesterol (mmol/L) and lipoprotein(a) (nmol/L) were measured enzymatically using the Beckman Coulter AU5800 analyzer.

Several variables were self-reported via questionnaire at recruitment. Smoking status was self-reported and was categorized as never, former, or current. Self-reported alcohol consumption was classified as: "Daily or almost daily", "Three or four times a week", "Once or twice a week", "One to three times a month", "Special occasions only", or "Never". Participants reported whether they had ever been diagnosed with diabetes by a doctor (yes/no). Physical activity was assessed using the International Physical Activity Questionnaire (IPAQ) and categorized as low, moderate, or high.[61] The Townsend Deprivation Index, based on the most recent census data, was calculated at baseline and used as a proxy for socioeconomic status. Participants reported their ethnic background, categorized as "White", "Asian", "Black", or "Other/multiple ethnicities". Participants were also asked about reproductive history, including age at menarche (in years), menopause status (yes/no), ever use of oral contraceptives (OCPs) (yes/no) and hormone replacement therapy (HRT) (yes/no), history of hysterectomy (yes/no) and/or bilateral oophorectomy (yes/no) including ages at surgery ("How old were you when you had your hysterectomy?" or "How old were you when you had both ovaries removed?"), the number of live births ("How many children have you given birth to? (Please include live births only)"), history of stillbirth ("How many stillbirths? (enter 0 if none)"), number of spontaneous miscarriages ("How many spontaneous miscarriages? (enter 0 if none)") and parity, which is the sum of all births and number of spontaneous miscarriages. Diagnoses of adenomyosis (N80.0), and polycystic ovarian syndrome (PCOS) (E28.2) were identified from linked health records, using the same procedure as for endometriosis and CVD outcomes.

Handling of missing values

Date variables defined by UKB Data-Coding 819 containing erroneous entries were treated as missing. In addition, responses such as “Prefer not to answer”, “Do not know”, or “Not sure” were also classified as missing values. If a date of endometriosis diagnosis was not available, the participant was still considered as having endometriosis. Missing data were handled using multiple imputation by chained equations (MICE), implemented via the *mice* package (v3.16.0).[64] The imputation model accounted for the data type of each variable, applying appropriate methods (i.e., predictive mean matching for continuous variables, logistic regression for binary variables, and polytomous regression for categorical variables with more than two levels). To avoid bias and multicollinearity, we restricted predictor relationships to prevent bidirectional imputation between highly correlated variables, limited to Pearson correlation coefficient threshold of ±0.7. In addition, CVD outcomes were not allowed to predict medication use, and vice versa. A total of 50 imputed datasets were generated. Each dataset was analyzed separately using the specified statistical model, and the results were combined using Rubin’s rules to obtain pooled estimates and confidence intervals (CIs).[65] The number of iterations was set to 10, and trace plots indicated satisfactory convergence.

# Supplementary tables

## Table S1. STROBE Checklist

| | Item No | Recommendation | Page No. |
|---|---|---|---|
| **Title and abstract** | 1 | (*a*) Indicate the study's design with a commonly used term in the title or the abstract | 2 |
| | | (*b*) Provide in the abstract an informative and balanced summary of what was done and what was found | 2 |
| **Introduction** | | | |
| Background/rationale | 2 | Explain the scientific background and rationale for the investigation being reported | 6 |
| Objectives | 3 | State specific objectives, including any prespecified hypotheses | 6 |
| **Methods** | | | |
| Study design | 4 | Present key elements of study design early in the paper | 7 |
| Setting | 5 | Describe the setting, locations, and relevant dates, including periods of recruitment, exposure, follow-up, and data collection | 7 |
| Participants | 6 | (*a*) *Cohort study*—Give the eligibility criteria, and the sources and methods of selection of participants. Describe methods of follow-up<br>*Case-control study*—Give the eligibility criteria, and the sources and methods of case ascertainment and control selection. Give the rationale for the choice of cases and controls<br>*Cross-sectional study*—Give the eligibility criteria, and the sources and methods of selection of participants | 7 |
| | | (*b*) *Cohort study*—For matched studies, give matching criteria and number of exposed and unexposed<br>*Case-control study*—For matched studies, give matching criteria and the number of controls per case | Not applicable |
| Variables | 7 | Clearly define all outcomes, exposures, predictors, potential confounders, and effect modifiers. Give diagnostic criteria, if applicable | 7 |
| Data sources/ measurement | 8* | For each variable of interest, give sources of data and details of methods of assessment (measurement). Describe comparability of assessment methods if there is more than one group | 7 |
| Bias | 9 | Describe any efforts to address potential sources of bias | 8 |
| Study size | 10 | Explain how the study size was arrived at | 7 |
| Quantitative variables | 11 | Explain how quantitative variables were handled in the analyses. If applicable, describe which groupings were chosen and why | 8 |
| Statistical methods | 12 | (*a*) Describe all statistical methods, including those used to control for confounding | 8 |
| | | (*b*) Describe any methods used to examine subgroups and interactions | 9 |
| | | (*c*) Explain how missing data were addressed | 37 |
| | | (*d*) *Cohort study*—If applicable, explain how loss to follow-up was addressed<br>*Case-control study*—If applicable, explain how matching of cases and controls was addressed<br>*Cross-sectional study*—If applicable, describe analytical methods taking account of sampling strategy | 8 |
| | | (*e*) Describe any sensitivity analyses | 10 |

| **Results** | | | **Page no.** |
|---|---|---|---|
| Participants | 13* | (a) Report numbers of individuals at each stage of study—eg numbers potentially eligible, examined for eligibility, confirmed eligible, included in the study, completing follow-up, and analysed | 11 |
| | | (b) Give reasons for non-participation at each stage | 32 |
| | | (c) Consider use of a flow diagram | 32 |
| Descriptive data | 14* | (a) Give characteristics of study participants (eg demographic, clinical, social) and information on exposures and potential confounders | 11 |
| | | (b) Indicate number of participants with missing data for each variable of interest | 30 |
| | | (c) *Cohort study*—Summarise follow-up time (eg, average and total amount) | 11,30 |
| Outcome data | 15* | *Cohort study*—Report numbers of outcome events or summary measures over time | 11 |
| | | *Case-control study*—Report numbers in each exposure category, or summary measures of exposure | |
| | | *Cross-sectional study*—Report numbers of outcome events or summary measures | |
| Main results | 16 | (*a*) Give unadjusted estimates and, if applicable, confounder-adjusted estimates and their precision (eg, 95% confidence interval). Make clear which confounders were adjusted for and why they were included | 11 |
| | | (*b*) Report category boundaries when continuous variables were categorized | 41 |
| | | (*c*) If relevant, consider translating estimates of relative risk into absolute risk for a meaningful time period | Not applicable |
| Other analyses | 17 | Report other analyses done—eg analyses of subgroups and interactions, and sensitivity analyses | 12 |
| **Discussion** | | | |
| Key results | 18 | Summarise key results with reference to study objectives | 14 |
| Limitations | 19 | Discuss limitations of the study, taking into account sources of potential bias or imprecision. Discuss both direction and magnitude of any potential bias | 18 |
| Interpretation | 20 | Give a cautious overall interpretation of results considering objectives, limitations, multiplicity of analyses, results from similar studies, and other relevant evidence | 19 |
| Generalisability | 21 | Discuss the generalisability (external validity) of the study results | 19 |
| **Other information** | | | |
| Funding | 22 | Give the source of funding and the role of the funders for the present study and, if applicable, for the original study on which the present article is based | 21 |

## Table S2. Sensitivity analyses within the mediation analysis

| | Hazard ratio or proportion mediated (95% confidence interval) | |
|---|---|---|
| **Mediator & effect** | **Classical approach** | **Only endometriosis diagnosed before hysterectomy** |
| **Hysterectomy with or without oophorectomy** | | |
| Direct Effect | 1.07 (0.99-1.15) | 1.08 (0.98-1.20) |
| Indirect Effect | 1.11 (0.99-1.24) | 1.07 (1.06-1.08) |
| Proportion Mediated | 62% (12;112%) | 45% (11;78%) |
| **Hysterectomy without oophorectomy** | | |
| Direct Effect | 1.15 (1.06-1.24) | 1.14 (1.03-1.25) |
| Indirect Effect | 1.03 (0.92-1.15) | 1.02 (1.01-1.02) |
| Proportion Mediated | 18% (-42;77%) | 12% (3;21%) |
| **Hysterectomy with oophorectomy** | | |
| Direct Effect | 1.13 (1.05-1.23) | 1.11 (1.01-1.23) |
| Indirect Effect | 1.05 (0.94-1.17) | 1.04 (1.02-1.05) |
| Proportion Mediated | 27% (-31;85%) | 25% (5;45%) |

This table summarizes two sensitivity analyses within our mediation framework. First, the classical mediation analysis, also known as the difference-in-coefficients method. Second, the counterfactual-based weighting approach restricted to endometriosis cases diagnosed before hysterectomy. Total, direct, and indirect effects of the exposure on the outcome are shown as hazard ratios, with the proportion mediated (%) illustrating the mediator's contribution to the exposure-outcome relationship.

## Supplementary figures

### Figure S1: Proportional Hazards assumption check

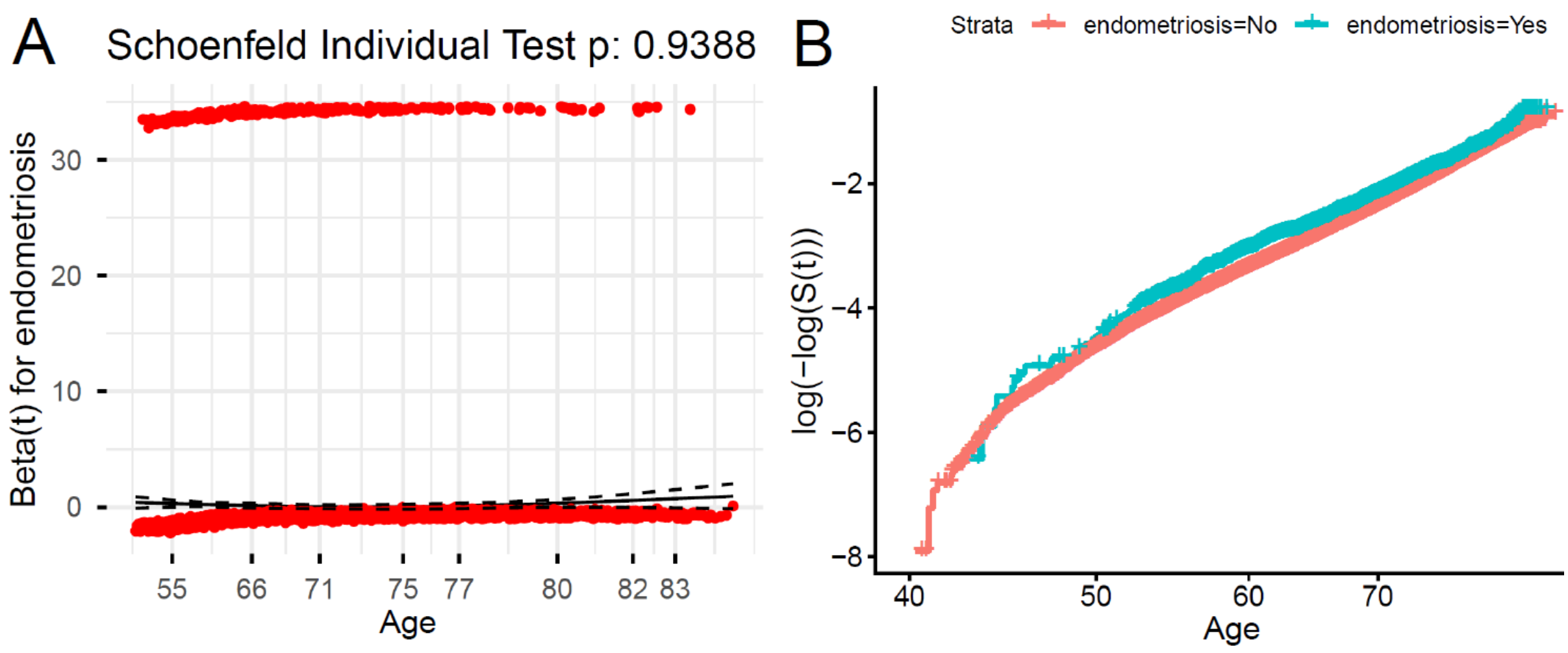


Assessment of the proportional hazards assumption. Panel A shows Schoenfeld residual plot for endometriosis with corresponding statistical tests from the fully adjusted model with composite CVD as outcome, and panel B log–log survival curves derived from Kaplan–Meier estimates for composite CVD, stratified by exposure group.

## Figure S2: Subgroup analysis

| Subgroup | Number of individuals | Number of events | HR (95% CI) | P−Value | Interaction P−Value |
|---|---|---|---|---|---|
| Age at baseline | | | | | 0.362 |
| ≥60 Years | 108881 | 14950 | 1.15 (1.03; 1.28) | 0.012 | |
| <60 Years | 155859 | 7333 | 1.23 (1.10; 1.37) | <0.001 | |
| Ever HRT use | | | | | 0.148 |
| No | 164911 | 10421 | 1.23 (1.08; 1.41) | 0.002 | |
| Yes | 99829 | 11862 | 1.09 (0.99; 1.20) | 0.070 | |
| Ever OCP use | | | | | 0.481 |
| No | 49562 | 5962 | 1.25 (1.06; 1.48) | 0.009 | |
| Yes | 215178 | 16321 | 1.17 (1.07; 1.27) | <0.001 | |
| History of spontaneous miscarriage | | | | | 0.632 |
| No | 209636 | 17347 | 1.19 (1.09; 1.31) | <0.001 | |
| Yes | 55104 | 4936 | 1.14 (0.97; 1.35) | 0.114 | |
| History of stillbirth | | | | | 0.202 |
| No | 258153 | 21461 | 1.20 (1.10; 1.29) | <0.001 | |
| Yes | 6587 | 822 | 0.84 (0.49; 1.47) | 0.549 | |
| Hysterectomy | | | | | 0.443 |
| No | 216183 | 16054 | 1.12 (0.97; 1.29) | 0.131 | |
| Yes | 48557 | 6229 | 1.05 (0.95; 1.15) | 0.354 | |
| Oophorectomy | | | | | 0.945 |
| No | 242611 | 19526 | 1.13 (1.02; 1.26) | 0.024 | |
| Yes | 22129 | 2757 | 1.13 (1.00; 1.27) | 0.051 | |
| Hysterectomy or oophorectomy | | | | | 0.537 |
| No | 215218 | 15948 | 1.11 (0.95; 1.29) | 0.176 | |
| Yes | 49522 | 6335 | 1.05 (0.95; 1.15) | 0.322 | |
| Menopausal status | | | | | 0.877 |
| No | 69192 | 1983 | 1.20 (0.91; 1.57) | 0.198 | |
| Yes | 195548 | 20300 | 1.17 (1.08; 1.27) | <0.001 | |
| Parity | | | | | 0.558 |
| 0 | 50075 | 3343 | 1.14 (0.96; 1.35) | 0.140 | |
| ≥1 | 214665 | 18940 | 1.20 (1.10; 1.31) | <0.001 | |
| PCOS | | | | | 0.735 |
| No | 263759 | 22210 | 1.18 (1.09; 1.28) | <0.001 | |
| Yes | 981 | 73 | 1.34 (0.60; 2.99) | 0.475 | |

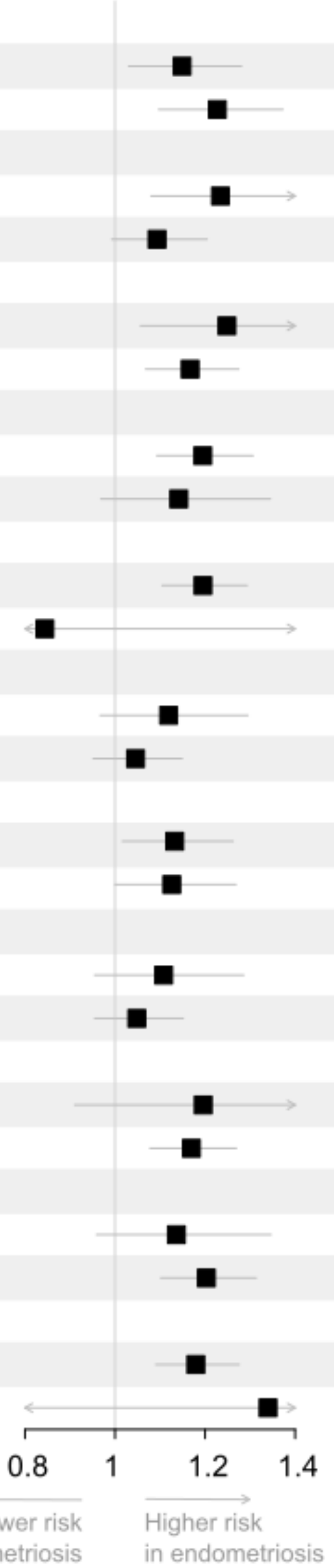


Subgroup analyses were conducted by including interaction terms between endometriosis and each subgroup variable in fully adjusted Cox models. Interaction term for age at baseline was calculated with continuous age variable. All models used the composite CVD endpoint and included covariates from the primary analysis. Abbreviations: HRT = hormone replacement therapy, PCOS = polycystic ovary syndrome, OCP = oral contraceptive pill, CVD = cardiovascular disease, HR = hazard ratio, CI = confidence interval. Number of individuals and number of events are based on first imputed dataset.

## Figure S3: Progressive adjustment

| Outcome and Adjustments | Number of events | HR (95% CI) | P−value |
|---|---|---|---|
| **Composite CVD** | 22283 | | |
| Age adjusted | | 1.19 (1.10; 1.28) | <0.001 |
| Above + conventional & emerging CVD risk factors | | 1.18 (1.09; 1.27) | <0.001 |
| Above + lifestyle & demographic risk factors | | 1.18 (1.09; 1.28) | <0.001 |
| Above + female−specific risk factors | | 1.18 (1.10; 1.28) | <0.001 |
| **Coronary heart disease** | 12975 | | |
| Age adjusted | | 1.25 (1.13; 1.38) | <0.001 |
| Above + conventional & emerging CVD risk factors | | 1.23 (1.12; 1.36) | <0.001 |
| Above + lifestyle & demographic risk factors | | 1.24 (1.12; 1.36) | <0.001 |
| Above + female−specific risk factors | | 1.24 (1.12; 1.37) | <0.001 |
| **Myocardial infarction** | 3274 | | |
| Age adjusted | | 1.07 (0.86; 1.32) | 0.556 |
| Above + conventional & emerging CVD risk factors | | 1.06 (0.86; 1.31) | 0.593 |
| Above + lifestyle & demographic risk factors | | 1.06 (0.86; 1.30) | 0.612 |
| Above + female−specific risk factors | | 1.06 (0.86; 1.30) | 0.611 |
| **Peripheral artery disease** | 1226 | | |
| Age adjusted | | 1.13 (0.80; 1.59) | 0.485 |
| Above + conventional & emerging CVD risk factors | | 1.14 (0.81; 1.60) | 0.458 |
| Above + lifestyle & demographic risk factors | | 1.14 (0.81; 1.61) | 0.442 |
| Above + female−specific risk factors | | 1.14 (0.81; 1.61) | 0.440 |
| **Stroke** | 8082 | | |
| Age adjusted | | 1.09 (0.95; 1.25) | 0.210 |
| Above + conventional & emerging CVD risk factors | | 1.09 (0.95; 1.25) | 0.214 |
| Above + lifestyle & demographic risk factors | | 1.10 (0.96; 1.26) | 0.177 |
| Above + female−specific risk factors | | 1.10 (0.96; 1.26) | 0.183 |
| **Hemorrhagic stroke** | 1181 | | |
| Age adjusted | | 1.03 (0.72; 1.46) | 0.888 |
| Above + conventional & emerging CVD risk factors | | 1.04 (0.73; 1.48) | 0.821 |
| Above + lifestyle & demographic risk factors | | 1.04 (0.73; 1.48) | 0.812 |
| Above + female−specific risk factors | | 1.04 (0.73; 1.48) | 0.821 |
| **Ischemic stroke** | 3282 | | |
| Age adjusted | | 1.09 (0.88; 1.35) | 0.427 |
| Above + conventional & emerging CVD risk factors | | 1.08 (0.88; 1.34) | 0.463 |
| Above + lifestyle & demographic risk factors | | 1.09 (0.88; 1.35) | 0.411 |
| Above + female−specific risk factors | | 1.09 (0.88; 1.35) | 0.409 |
| **Cardiovascular mortality** | 2388 | | |
| Age adjusted | | 0.84 (0.63; 1.12) | 0.239 |
| Above + conventional & emerging CVD risk factors | | 0.84 (0.62; 1.12) | 0.232 |
| Above + lifestyle & demographic risk factors | | 0.85 (0.63; 1.14) | 0.270 |
| Above + female−specific risk factors | | 0.85 (0.63; 1.13) | 0.265 |
| **All−cause mortality** | 16572 | | |
| Age adjusted | | 0.88 (0.79; 0.98) | 0.020 |
| Above + conventional & emerging CVD risk factors | | 0.88 (0.79; 0.98) | 0.017 |
| Above + lifestyle & demographic risk factors | | 0.88 (0.80; 0.98) | 0.022 |
| Above + female−specific risk factors | | 0.88 (0.80; 0.98) | 0.022 |

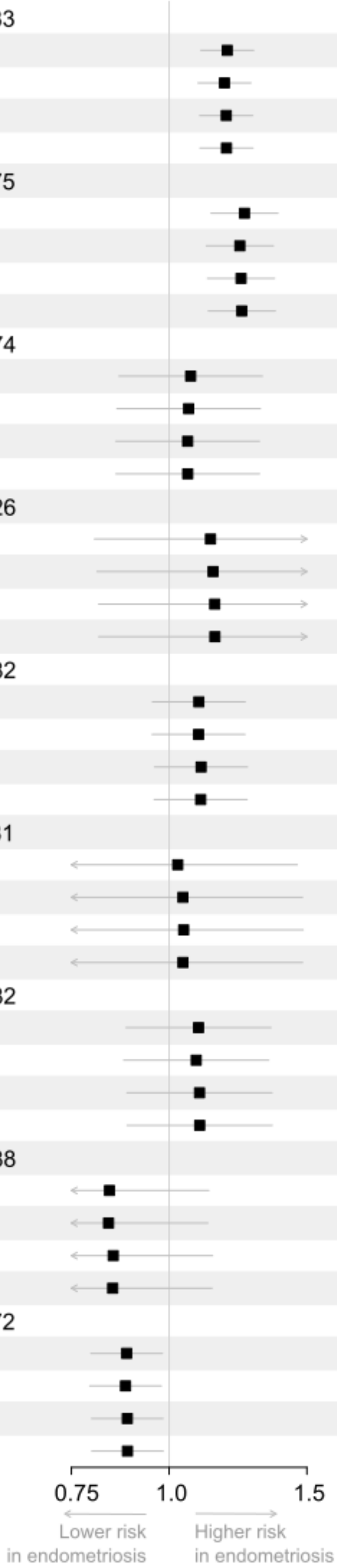


Forest plot showing associations between endometriosis and events across increasing adjustment levels. Hazard ratios with 95% confidence intervals were estimated using Cox proportional hazards models with attained age as the timescale. The model with conventional and emerging CVD risk factors was adjusted for BMI, smoking status, diabetes, LDL-cholesterol, systolic blood pressure, and lipoprotein(a). The lifestyle and demographic model additionally included alcohol use, physical activity, ethnicity, and the Townsend Deprivation Index. The female-specific model was further adjusted for age at menarche and oral contraceptive use, with each step building on the preceding models. Abbreviations: HRT = hormone replacement therapy, PCOS = polycystic ovary syndrome, OCP = oral contraceptive pill, CVD = cardiovascular disease, HR = hazard ratio, CI = confidence interval. In the age adjusted models, age adjustment is achieved by using attained age as the underlying time scale in the survival models, rather than by including age as an explicit covariate in the regression.

## Figure S4: Sensitivity analyses

| Sensitivity Analysis | HR (95% CI) | P−value |
|---|---|---|
| Non−self−reported endometriosis | 1.18 (1.07; 1.30) | 0.001 |
| Endometriosis diagnosis before hysterectomy | 1.15 (1.05; 1.27) | 0.004 |
| Exclude coexisting adenomyosis | 1.16 (1.06; 1.26) | 0.001 |
| Time to event as timescale | 1.19 (1.10; 1.28) | <0.001 |
| Censoring event within 3 years of endometriosis | 1.18 (1.09; 1.27) | <0.001 |
| Fine−Gray subdistribution for competing events | 1.18 (1.10; 1.26) | <0.001 |

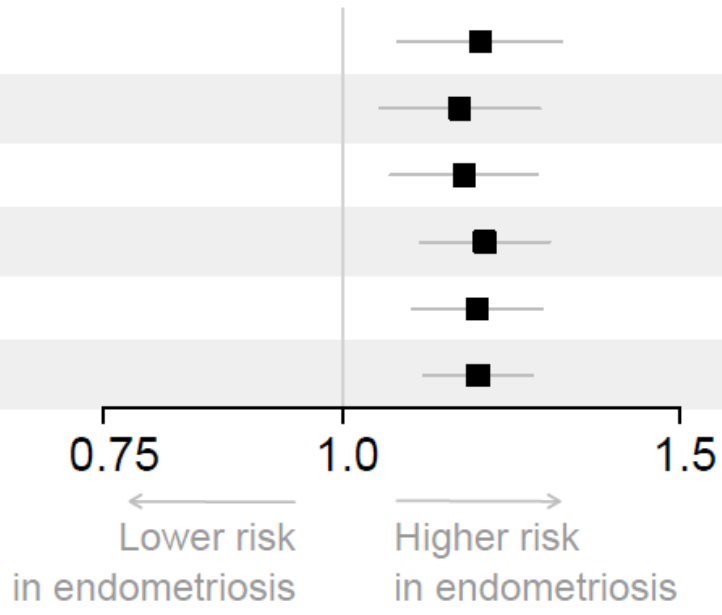


Forest plot shows the association between endometriosis and composite CVD across several sensitivity analyses. This plot displays hazard ratios with 95% confidence intervals from Cox proportional hazards models with full adjustment and attained age as timescale. Full adjustment includes conventional and emerging CVD risk factors (BMI, smoking status, diabetes, LDL-cholesterol, systolic blood pressure, and lipoprotein(a)), lifestyle and demographic factors (alcohol use, physical activity, ethnicity, and the Townsend Deprivation Index), and female-specific factors (age at menarche and ever OCP use). Abbreviations: CVD = cardiovascular disease, HR = hazard ratio, CI = confidence interval.

## Figure S5: Complete case analysis

| Cardiovascular Event and Adjustments | Number of individuals | Number of events | HR (95% CI) | P−Value |
|---|---|---|---|---|
| **Composite CVD** | | | | |
| Age adjusted | 264740 | 22283 | 1.19 (1.10; 1.28) | <0.001 |
| Above + conventional & emerging CVD risk factors | 184874 | 15106 | 1.15 (1.05; 1.27) | 0.003 |
| Above + lifestyle & demographic risk factors | 137556 | 10270 | 1.14 (1.01; 1.28) | 0.030 |
| Above + female−specific risk factors | 134182 | 10007 | 1.15 (1.02; 1.29) | 0.022 |
| **Coronary heart disease** | | | | |
| Age adjusted | 264740 | 12975 | 1.25 (1.13; 1.38) | <0.001 |
| Above + conventional & emerging CVD risk factors | 184874 | 8766 | 1.19 (1.05; 1.34) | 0.006 |
| Above + lifestyle & demographic risk factors | 137556 | 5969 | 1.14 (0.98; 1.33) | 0.079 |
| Above + female−specific risk factors | 134182 | 5819 | 1.15 (0.99; 1.34) | 0.061 |
| **Myocardial infarction** | | | | |
| Age adjusted | 264740 | 3274 | 1.07 (0.86; 1.32) | 0.556 |
| Above + conventional & emerging CVD risk factors | 184874 | 2177 | 1.00 (0.77; 1.31) | 0.972 |
| Above + lifestyle & demographic risk factors | 137556 | 1501 | 0.91 (0.66; 1.27) | 0.592 |
| Above + female−specific risk factors | 134182 | 1454 | 0.93 (0.67; 1.30) | 0.673 |
| **Peripheral artery disease** | | | | |
| Age adjusted | 264740 | 1226 | 1.13 (0.80; 1.59) | 0.485 |
| Above + conventional & emerging CVD risk factors | 184874 | 783 | 1.14 (0.74; 1.74) | 0.556 |
| Above + lifestyle & demographic risk factors | 137556 | 523 | 1.15 (0.69; 1.92) | 0.595 |
| Above + female−specific risk factors | 134182 | 507 | 1.10 (0.65; 1.87) | 0.727 |
| **Stroke** | | | | |
| Age adjusted | 264740 | 8082 | 1.09 (0.95; 1.25) | 0.210 |
| Above + conventional & emerging CVD risk factors | 184874 | 5557 | 1.10 (0.94; 1.30) | 0.232 |
| Above + lifestyle & demographic risk factors | 137556 | 3778 | 1.12 (0.92; 1.36) | 0.246 |
| Above + female−specific risk factors | 134182 | 3681 | 1.14 (0.94; 1.38) | 0.197 |
| **Hemorrhagic stroke** | | | | |
| Age adjusted | 264740 | 1181 | 1.03 (0.72; 1.46) | 0.888 |
| Above + conventional & emerging CVD risk factors | 184874 | 836 | 0.91 (0.59; 1.42) | 0.690 |
| Above + lifestyle & demographic risk factors | 137556 | 595 | 1.01 (0.61; 1.65) | 0.984 |
| Above + female−specific risk factors | 134182 | 579 | 1.02 (0.62; 1.69) | 0.924 |
| **Ischemic stroke** | | | | |
| Age adjusted | 264740 | 3282 | 1.09 (0.88; 1.35) | 0.427 |
| Above + conventional & emerging CVD risk factors | 184874 | 2219 | 1.10 (0.85; 1.42) | 0.483 |
| Above + lifestyle & demographic risk factors | 137556 | 1519 | 1.09 (0.80; 1.49) | 0.583 |
| Above + female−specific risk factors | 134182 | 1485 | 1.10 (0.81; 1.50) | 0.547 |
| **Cardiovascular mortality** | | | | |
| Age adjusted | 264740 | 2388 | 0.84 (0.63; 1.12) | 0.239 |
| Above + conventional & emerging CVD risk factors | 184874 | 1577 | 0.84 (0.59; 1.20) | 0.340 |
| Above + lifestyle & demographic risk factors | 137556 | 1014 | 0.89 (0.58; 1.38) | 0.611 |
| Above + female−specific risk factors | 134182 | 990 | 0.90 (0.58; 1.39) | 0.640 |
| **All−cause mortality** | | | | |
| Age adjusted | 264740 | 16572 | 0.88 (0.79; 0.98) | 0.020 |
| Above + conventional & emerging CVD risk factors | 184874 | 11078 | 0.94 (0.83; 1.07) | 0.359 |
| Above + lifestyle & demographic risk factors | 137556 | 7530 | 0.94 (0.81; 1.09) | 0.406 |
| Above + female−specific risk factors | 134182 | 7305 | 0.95 (0.81; 1.10) | 0.468 |

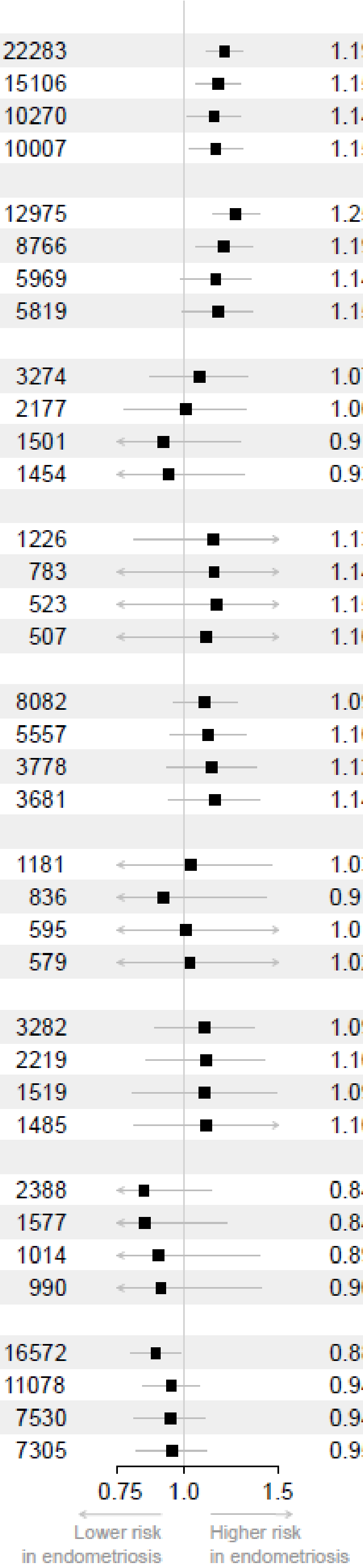


Forest plot showing associations between endometriosis and cardiovascular events across increasing adjustment levels. Hazard ratios with 95% confidence intervals were estimated using Cox proportional hazards models with attained age as the timescale. The model with conventional and emerging CVD risk factors was adjusted for BMI, smoking status, diabetes, LDL-cholesterol, systolic blood pressure, and lipoprotein(a). The lifestyle and demographic model additionally included alcohol use, physical activity, ethnicity, and the Townsend Deprivation Index. The female-specific model was further adjusted for age at menarche and oral contraceptive use, with each step building on the preceding models. This analysis was restricted to participants with complete data on all covariates. Abbreviations: HRT = hormone replacement therapy, PCOS = polycystic ovary syndrome, OCP = oral contraceptive pill, CVD = cardiovascular disease, HR = hazard ratio, CI = confidence interval. In the age adjusted models, age

adjustment is achieved by using attained age as the underlying time scale in the survival models, rather than by including age as an explicit covariate in the regression.

## Figure S6: Directed acyclic graph

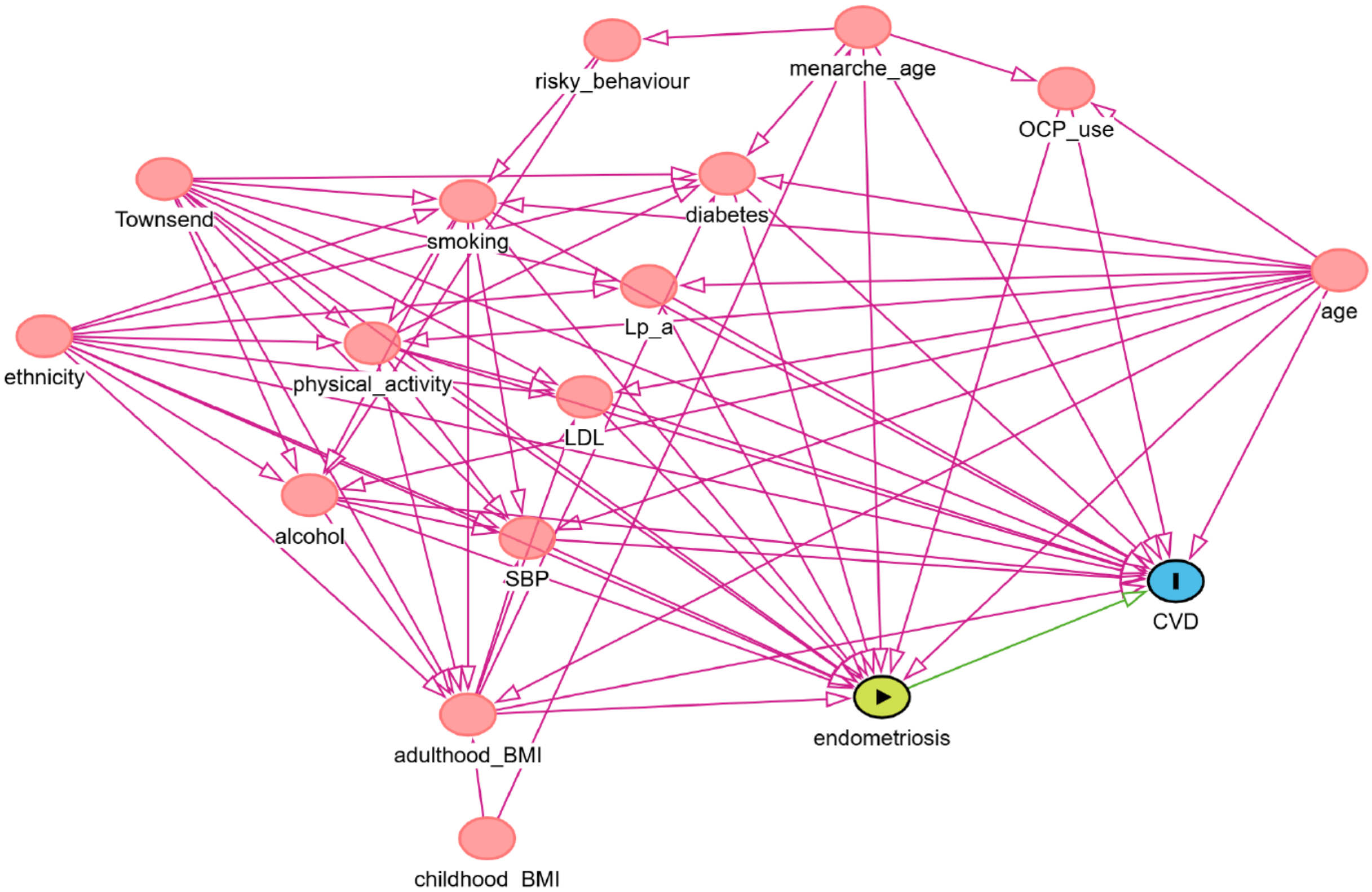


Directed acyclic graph illustrating the assumed causal structure for the association between endometriosis and cardiovascular disease, used to guide confounder selection. Created with DAGitty v3.1[66]. Abbreviations: OCP = oral contraceptive pill, Lp_a = lipoprotein(a), LDL = low-density lipoprotein cholesterol, SBP = systolic blood pressure, BMI = body mass index, CVD = cardiovascular disease.
